# Transcutaneous auricular vagus nerve stimulation attenuates depressive-like behaviors via enhancing neuroplasticity and regulating the ALK5/Smad2/3/Gadd45β signaling pathway in rats with post-stroke depression

**DOI:** 10.3389/fimmu.2026.1755707

**Published:** 2026-05-11

**Authors:** Qinghuan Yang, You Zhou, Hao Tang, Jingxi Ma, Gongwei Jia, Yangmei Chen, Lingchuan Niu, Jiani Li

**Affiliations:** 1 Department of Neurology, The Second Affiliated Hospital of Chongqing Medical University, Chongqing, China; 2 Department of Neurology, The First Affiliated Hospital of Chongqing Medical University, Chongqing, China; 3 Department of Critical Care Medicine, The Second Affiliated Hospital of Chongqing Medical University, Chongqing, China; 4 Department of Neurology, Chongqing General Hospital, Chongqing, China; 5 Chongqing University, Chongqing, China; 6 Department of Rehabilitation, The Second Affiliated Hospital of Chongqing Medical University, Chongqing, China

**Keywords:** ALK5, depression-like behavior, GADD45β, neuroplasticity, post-stroke depression, transcutaneous vagus nerve stimulation

## Abstract

**Introduction:**

Post-stroke depression (PSD) adversely affects neurological functional recovery in patients. Transcutaneous auricular vagus nerve stimulation (ta-VNS) has demonstrated antidepressant potential. As the receptor ALK5 may regulate neuroplasticity by modulating the Smad2/3 and Gadd45β signaling pathways, we hypothesized that ta-VNS alleviates PSD by activating this pathway. This study aimed to evaluate the therapeutic effects of ta-VNS on a PSD rat model and to investigate the involvement of the ALK5/Smad2/3/Gadd45β signaling pathway in mediating these effects.

**Methods:**

A PSD rat model was established by combining middle cerebral artery occlusion (MCAO) with chronic unpredictable mild stress (CUMS). To investigate the underlying mechanisms, ALK5 expression was knocked down in the right prefrontal cortex (PFC) via AAV-shALK5 injection, followed by a 14-day ta-VNS treatment. Depression-like behaviors were evaluated using the sucrose preference, forced swimming, and open-field tests. Neuroprotection was assessed through hematoxylin–eosin, Nissl, and TUNEL staining. Neurotransmitter expression was measured by enzyme-linked immunosorbent assay (ELISA). Neurogenesis, along with axonal, dendritic, and synaptic plasticity, was assessed by immunostaining and Western blot analysis of DCX, Nestin, NF-200, GAP-43, MAP-2, and PSD95, SYN. Additionally, dendritic morphology was visualized by Golgi-Cox staining, and in addition, synaptic ultrastructure was characterized using transmission electron microscopy. Protein levels of the ALK5/Smad2/3/Gadd45β pathway were analyzed by Western blot.

**Results:**

Ta-VNS treatment restored the PSD-induced downregulation of ALK5. Furthermore, the beneficial effects of ta-VNS—including the amelioration of depressive-like behaviors, provision of neuroprotection, upregulation of serotonin (5-HT) and dopamine (DA) expression, promotion of neurogenesis, and enhancement of neuroplasticity—were all abolished upon ALK5 knockdown. Finally, ta-VNS was found to activate the Smad2/3/Gadd45β signaling pathway in an ALK5-dependent manner.

**Conclusion:**

Our findings demonstrate that ta-VNS ameliorates depressive-like behaviors and enhances neuroplasticity in PSD by activating the ALK5/Smad2/3/Gadd45β signaling pathway in the PFC. These results elucidate a novel molecular mechanism underlying the therapeutic effects of ta-VNS, highlighting its potential as a treatment strategy for PSD.

## Graphical Abstract

**Figure:**
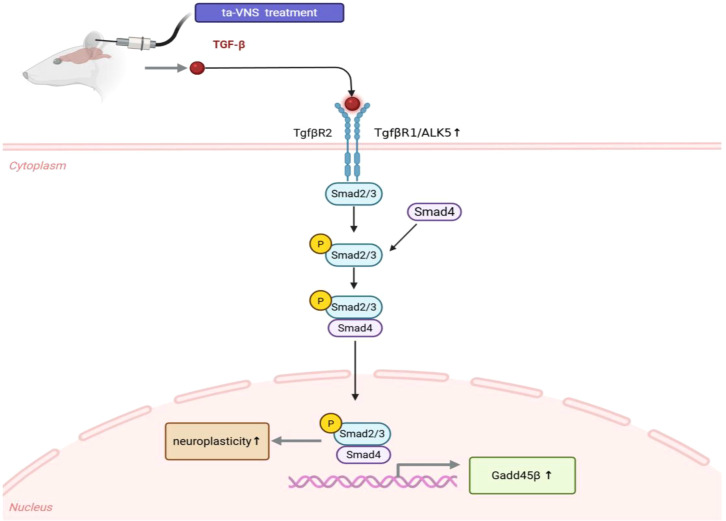
Schematic diagram illustrating that ta-VNS treatment upregulates ALK5 expression, thereby triggering a signaling cascade involving Smad2/3 phosphorylation. The phosphorylated Smad2/3 then binds to Smad4, and the resulting complex translocates into the nucleus to drive the transcription of Gadd45β, ultimately promoting neuroplasticity and thereby improving depressive-like behaviors.

## 1 Introduction

With approximately 7 million fatalities, stroke persisted as the second foremost cause of death among non-communicable diseases, based on the newest stroke burden findings from the Global Burden of Disease (GBD) 2021 study (1). It is also a major contributor to long-term disability, significantly reducing healthy life-years as a result of serious after-effects such as paralysis, speech impairments, swallowing difficulties, epilepsy, and cognitive deficits (2–5). These severe symptoms and functional limitations pose considerable health challenges for stroke survivors, who also face an increased risk of developing psychological conditions. Among these, depression is one of the most frequent complications, affecting an estimated 30%–33% of patients (6). Those with post-stroke depression (PSD) often report feelings of anxiety, hopelessness, social withdrawal, and sleep disturbances, all of which can hinder daily functioning and recovery efforts (7). Previous studies (7–9) indicate that PSD involves disrupted neuroplasticity in the prefrontal cortex (PFC) and its connections to other brain regions; this disruption is known to increase the risk of depression in humans and cause depression-like symptoms in rodents. However, the exact pathophysiological pathways are still unclear.

Vagus nerve stimulation (VNS) represents a promising therapeutic approach with demonstrated potential in addressing a range of medical and neuropsychiatric conditions. Initially recognized for its efficacy in treating epilepsy, VNS was subsequently approved for use in treatment-resistant depression (10). However, the adoption of VNS has been constrained during its initial decades of use, primarily due to the invasive surgery and high costs associated with implanted devices. Recent advances in non-invasive VNS techniques have now enabled researchers to investigate the effects of vagal nerve modulation with greater precision and accessibility (11). Our previous research found that transcutaneous vagus nerve stimulation (ta-VNS) can improve the psychiatric symptoms of patients with PSD (12). The engagement of neuromodulatory networks involved in neuroplasticity provides a mechanism by which VNS probably facilitates brain repair (13). In a rat model of middle cerebral artery occlusion (MCAO), we found that (14) ta-VNS improved functional recovery. These benefits, which included enhanced axonal plasticity and regeneration, were mediated by the cholinergic anti-inflammatory pathway. Recently, evidence has emerged indicating that ta-VNS treatment may have the potential to regulate cortical neuroplasticity and facilitate the recovery of depression (6, 15). Nevertheless, the role of ta-VNS treatment on neuroplasticity following PSD and the underlying mechanisms remain poorly understood.

Research (16) has shown that transforming growth factor-beta (TGF-β) is critical for central nervous system development, regulating processes from early embryogenesis to adult neurogenesis. As one of its principal receptors, activin-receptor-like-kinase-5 (ALK5) has also attracted considerable attention. Ungefroren et al. found (17) that ALK5/TGF-β type I receptors regulate the expression of the growth arrest and DNA damage-inducible protein β (Gadd45β). Research (18) indicates that Gadd45β contributes to the support and maintenance of the nervous system. The Smad family mediates TGF-β signal transduction. Smad2/3, in particular, is an essential component of this pathway, facilitating the translocation of ALK5 from cell surface receptors to the nucleus (19). Our previous investigation (20) suggested that ALK5 modulates neuroplasticity through the Smad2/3/Gadd45β signaling pathway. Interestingly, our preliminary experiments revealed that ta-VNS intervention in a PSD rat model upregulates ALK5 expression through activation of the cholinergic anti-inflammatory pathway. These findings support the hypothesis that ta-VNS treatment has a beneficial effect on alleviating depressive symptoms following stroke in rats. The underlying mechanism may involve the upregulation of the ALK5/Smad2/3/Gadd45β pathway to promote neuroplasticity.

In summary, this study aimed to investigate whether ta-VNS improves depressive symptoms following stroke and to determine if this effect is mediated through the ALK5/Smad2/3/Gadd45β signaling pathway by enhancing neuroplasticity.

## 2 Materials and methods

### 2.1 Animals and experimental design

A total of 296 of adult male Sprague-Dawley rats (weight range: 230–280 g) were used in this study. The animals were obtained from the Animal Experiment Center of Chongqing Medical University. Every effort was made to minimize both the number of animals used and any potential distress experienced during the experiments. The rats were housed in a pathogen-free facility at the Animal Experiment Center of Chongqing Medical University. Ambient conditions were maintained at a temperature of 22 ± 1°C and approximately 60% relative humidity, under a 12-h light/12-h dark cycle to mimic natural circadian rhythms. Animals were group-housed with five rats per cage and provided with ad libitum access to food and water.

**Figure 1:**
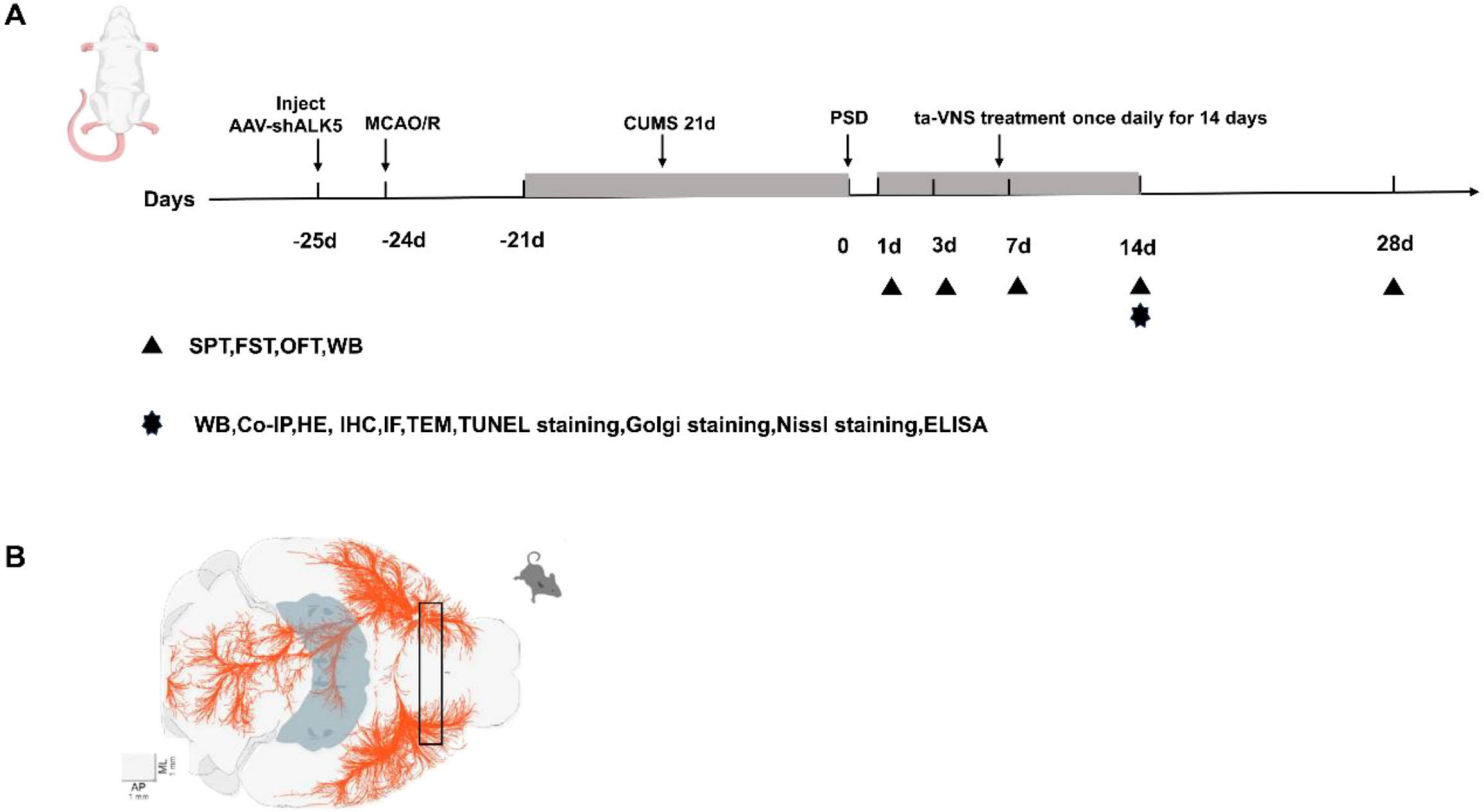
**(A)** The timeline diagram illustrates the experimental protocols. **(B)** Schematic diagram of rat prefrontal cortex sample collection.

The study was divided into two separate parts, and rats were randomly assigned to different groups. To investigate ALK5 expression in the prefrontal cortex, the first part of the study included 6 groups: (1) sham group, (2) PSD group (1d), (3) PSD group (3d), (4) PSD group (7d), (5) PSD group (14d), (6) PSD group (28d). To explore the role of ALK5 in ta-VNS-mediated neuroplasticity, we used five groups in the second part: (1) sham group, (2) PSD group, (3) PSD+ta-VNS group (4) PSD+ta-VNS+AAV-Scramble group, and (5) PSD+ta-VNS+AAV-shALK5 group. A flow diagram of the study is presented below (**Figures 1A, B**).

### 2.2 Establishment of PSD modeling

A right middle cerebral artery occlusion/reperfusion (MCAO/R) model was first established. Briefly, rats were anesthetized using 2.5% isoflurane and the surgical site was disinfected. The common carotid artery (CCA), internal carotid artery (ICA), and external carotid artery (ECA) were carefully exposed. The CCA was clamped, and the ECA was permanently ligated and transected. A paraffin-coated nylon monofilament was then carefully advanced until laser Doppler flowmetry (LDF) confirmed successful middle cerebral artery (MCA) occlusion, evidenced by a rapid drop in ipsilateral blood flow to 20%–30% of baseline levels. Following a 120-min occlusion period, the suture was removed to initiate cerebral reperfusion. Proper and timely restoration of blood flow was subsequently verified by LDF monitoring over a 30-min period (21).Arterial blood pressure, blood gases, and heart rate were monitored continuously, as described previously (14). Postoperatively, animals were kept in a temperature-controlled environment until they regained consciousness, after which they were returned to their standard housing. To prevent infection, penicillin (40,000 U/day) was administered via intraperitoneal injection for three consecutive days. Neurological deficits were evaluated using the Longa 5-point scale. Rats exhibiting neurological deficit scores of ≥1 but <4 on the Longa scale were selected for the study. We ensured that there was no statistically significant difference in the baseline scores among the MCAO/R model groups following Longa score assessment. Following a 3-day recovery period in individual cages, a chronic unpredictable mild stress (CUMS) protocol was initiated. Rats designated to the PSD and treatment groups were individually housed and subjected daily to a randomized sequence of one of eight distinct stressors over a 21-day period (22). The stress paradigms consisted of cage shaking (1 shake/s for 5 min), cage tilting at 45° (15 h), 24-h food and water deprivation, cold water swimming at 4°C (5 min), tail pinching (1 min), exposure to damp bedding (10 h), continuous overnight illumination (12 h), and physical restraint (2 h). Sham-operated rats received the same procedure, but the filament was advanced only 5 mm and did not occlude the middle cerebral artery.

### 2.3 ta-VNS treatment

Based on our previously established protocol (14), rats in the ta-VNS group received ta-VNS via two acupuncture needles connected to a Grass Model S48 stimulator. The needles were inserted superficially (0.5–1 mm beneath the skin) in the left cavum concha. Stimulation was delivered using the following parameters: 30-s trains of 0.5-ms square-wave pulses at 20 Hz, with a current intensity of 0.5 mA, applied every 5 min over a 1-h session. Treatment was initiated 1 day after PSD induction and administered once daily until sacrifice. Rats in the sham stimulation group underwent an identical procedure, including needle insertion, but without electrical stimulation.

### 2.4 Administration of adeno-associated virus

To knock down ALK5 expression, we used a short hairpin RNA (shRNA) targeting the sequence GCCATAACCGCACTGTCATTC within the Tgfbr1 gene (which encodes ALK5). Recombinant adeno-associated virus (AAV) vectors expressing this shRNA and an EGFP reporter under the control of a U6 and CMV promoter, respectively, were commercially packaged and obtained from OBiO Technology Co., Ltd (Shanghai, China), with a titer of 4.44 × 10¹² vg/mL. AAV vectors expressing a scrambled shRNA and EGFP from the same company were used as the control. One day prior to MCAO/R induction, AAV-shALK5 was stereotactically injected into the site within the right PFC (2 μL per site) using the following coordinates relative to bregma: anteroposterior (AP) +2.8 mm, mediolateral (ML)+0.6 mm, and dorsoventral (DV) −3.2 mm, based on the brain map of rats. The injection rate was 0.2 μL/min (21). Following injection, the needle was left in place for an additional 8 min to minimize backflow before being slowly withdrawn.

### 2.5 Behavioral tests

We assessed depression-like behaviors at five time points (1, 3, 7, 14, and 28 days post-PSD) using a behavioral battery that included the sucrose preference test (SPT), forced swimming test (FST), open-field test (OFT), and novel object recognition (NOR).

#### 2.5.1 SPT

SPT is used to evaluate an animal’s hedonic capacity based on its voluntary intake of a sucrose solution. In this test, a higher consumption of the sucrose solution is interpreted as a sign of better recovery. The experimental steps are as follows: The rats were first habituated to two bottles of 1% sucrose solution for 24 h. Subsequently, they were presented with one bottle of 1% sucrose solution and one bottle of water for an additional 24-h period. For the formal testing phase, following a 24-h water deprivation period, the rats were given two identical bottles—one containing 1% sucrose solution and the other filled with water. To eliminate positional bias, the bottles’ locations were randomly alternated. Sucrose and water consumption were measured over an 8-h period (23), and the experimental data were collected on the 1st, 3rd, 7th, 14th, and 28th days following PSD modeling.

#### 2.5.2 FST

The FST evaluates an animal’s response to acute stress. Increased immobility time in the test correlates with a higher level of negative emotional states. The experimental steps are as follows: The rats were placed in a cylindrical water-filled bucket (50 cm in diameter, 25 cm deep) for the forced swim test. Following a 1-min acclimatization period, their immobility time was recorded over the subsequent 6-min observation window (24). The collection time of the experimental data is the same as above.

#### 2.5.3 OFT

Movement reduction in the OFT (time spent in the central area, distance traveled) reflects reduced activity and curiosity, which are common symptoms of depression. The experimental steps are as follows: The rats were placed in the center of an open square arena (100 cm × 100 cm × 40 cm) and allowed to explore freely for 5 min. The floor of the arena was divided into three concentric rectangular zones: the central zone, middle zone, and external zone. An infrared camera recorded the rats’ behavior, and the total distance traveled as well as time spent in the central zone were automatically analyzed using tracking software. After each trial, the arena was thoroughly cleaned with 75% ethanol to remove residual odors (25). The collection time of the experimental data is the same as above.

#### 2.5.4 NOR

To assess learning and memory in rodents, the NOR test was employed, following established protocols (26). During the habituation phase, rats were placed individually in a square opaque arena (20 cm × 20 cm) containing two identical objects. Each animal was allowed 5 min to acclimate to the environment before being returned to its home cage. In the subsequent testing phase, one of the familiar objects was replaced with a novel one differing in material, shape, and color. The rat was then reintroduced to the arena for another 5-min exploration period. Behavioral data, including the duration of sniffing or climbing each object, were recorded and analyzed using Smart software. A discrimination ratio was subsequently calculated as (Time spent with novel object – Time spent with familiar object)/(Time spent with novel object + Time spent with familiar object).

### 2.6 Western blotting

The rats were sacrificed, and the right PFC tissues were rapidly removed on ice and preserved at −80°C. An appropriate amount of tissue was collected and lysed by RIPA lysis buffer containing protease and phosphatase inhibitor. The mixture was then centrifuged at 12,000 rpm for 20 min. The BCA kit (Beyotime, China) was used to measure the protein concentration. After electrophoresis, the separated proteins were transferred onto polyvinylidene difluoride (PVDF) membranes (IPVH00010, Millipore, USA) and blocked with 5% bovine serum albumin. The membranes were then incubated overnight at 4°C with the following primary antibodies: anti-ALK5 (1:1,000, EPR20923-13, Abcam, USA), anti-p-Smad2/3 (1:1,000, #8828, Cell Signaling Technology, USA), anti-Smad2/3 (1:1,000, #3102, Cell Signaling Technology), anti-Gadd45β (1:1,000, ab230646, Abcam, USA), anti-doublecortin (DCX) (1:500, 13925-1-AP, Proteintech, China), anti-Nestin (1:500, 19483-1-AP,Proteintech,China), anti-synaptophysin (SYN) (1:10,000, 17785-1-AP,Proteintech,China), anti-postsynaptic density protein95 (PSD95) (1:2,000, 20665-1-AP, Proteintech, China), anti-β-Actin (1:4,000, 20536-1-AP, Proteintech, China), and anti-GAPDH (1:4,000, 10494-1-AP, Proteintech, China). GAPDH and β-actin were used as a loading control. After washing with TBST, the membranes were incubated with Goat anti-rabbit IgG (1:4,000, A23920, Abbkine, USA) for 1 h at room temperature. Subsequently, images were acquired with a Fusion FX5 analysis system (Vilber Lourmat, F-77601 Marne-la-Vall’ee Cedex 3, France) and quantified using Quantity One software (Bio-Rad Laboratories, USA).

### 2.7 Immunofluorescence assay

Rat PFC tissue collection and frozen section preparation were performed according to the protocol established by Liu et al (21). Briefly, the frozen sections were permeabilized with 0.4% Triton X-100, followed by antigen retrieval using sodium citrate. After three washes with phosphate-buffered saline (PBS), the sections were blocked with 5% normal goat serum for 1 h at room temperature. Subsequently, the sections were incubated overnight at 4 °C with the following primary antibodies: Anti-ALK5 (1:200, 30117-1-AP, Proteintech, China), Anti-Gadd45β (1:100, PA5-100741, Thermo Fisher, USA), Anti-NeuN (1:300, ABN78, MilliporeSigma, USA), Anti-GFAP (1:200, sc-51908, Santa Cruz Biotechnology, USA), Anti-Iba1 (1:4,000, EPR16588, Abcam, Cambridge, UK), Anti-DCX (1:100, 13925-1-AP, Proteintech, China), Anti-Nestin (1:100, 19483-1-AP, Proteintech, China), and Anti-MAP2 (1:100, #4542,CST, USA). The next day, the sections were washed with PBS and incubated for 1 h at 37 °C in the dark with secondary antibodies: Goat anti-rabbit IgG (1:400, A23920, Abbkine, USA) and Goat anti-mouse IgG (1:400, A23910, Abbkine, USA). Finally, the sections were counterstained with DAPI for nuclear visualization. Fluorescence images were acquired using a fluorescence microscope.

### 2.8 TUNEL staining

The brains were fixed by submersion in 10% formalin overnight at 4 °C. After fixation, they were washed with PBS and embedded in paraffin. The embedded tissues were then processed into 5-μm-thick sections. Following the manufacturer’s instructions (G1502-50T, Servicebio, Wuhan, China), the sections were incubated with TUNEL staining reagents. Stained images were acquired using a fluorescence microscope and NIS-Element Viewer software. The TUNEL-positive rate in the PFC was quantified using ImageJ software.

### 2.9 Immunohistochemistry

The immunohistochemistry (IHC) procedure, including tissue acquisition and section preparation, was performed following the methodology described by Liu et al (21). Tissue sections were blocked with 5% bovine serum albumin (BSA) at 37 °C for 1 h and then incubated overnight at 4 °C with the following primary antibodies: Anti-neurofilament antibody (1:200, 18934-1-AP, Proteintech, China) and rabbit monoclonal anti-growth-associated protein 43 (GAP43) (1:200, 16971-1-AP, Proteintech, China). The following day, sections were incubated with goat anti-rabbit IgG for 30 min at 37 °C. Positive immunoreactivity was visualized using 3,3'-diaminobenzidine (DAB) (Proteintech, China). Images were captured using a LEICA DM600B automated microscope and analyzed with FIJI software. Protein expression levels were quantified as the mean optical density (integrated optical density normalized to the relevant area).

### 2.10 Hematoxylin and eosin staining protocol for rat brain tissue

Paraffin-embedded rat brain tissue sections were first dewaxed and then dehydrated through a graded alcohol series (1 min per step). After rehydration in PBS for 1 min, the sections were stained with hematoxylin for 20 min to visualize nuclei, followed by a 20-min rinse under running tap water to enhance nuclear bluing. Subsequently, cytoplasmic staining was performed using eosin for 2 min, after which excess dye was removed by PBS rinsing. The sections were then dehydrated again through graded alcohols, cleared, and mounted with neutral resin. Finally, cortex tissue structures were examined under a light microscope to assess morphological changes.

### 2.11 Nissl staining

The dewaxed sections were rinsed three times with water and incubated in a nylon staining solution for 20 min at 60 °C. A further three water washes were performed, followed by drying at 60 °C. The sections were rendered transparent through xylene treatment and permanently sealed with neutral gum. Microscopic examination was conducted to evaluate neuronal damage in the rat brain tissue. Quantification of Nissl bodies was carried out by two independent, blinded investigators, and their average counts were adopted for each slice.

### 2.12 Golgi-Cox staining and dendritic analysis

Brain tissue samples were processed using the Hito Golgi-Cox OptimStain™ Kit (Hitobiotec Corp., USA) following the manufacturer’s protocol. Immediately following extraction, tissues were immersed in an equal-volume mixture of solutions 1 and 2 and stored in light-protected conditions at room temperature for 14 days. Subsequently, tissues were transferred to solution 3 for an additional 3 days under identical storage conditions. For sectioning, impregnated tissues were cryoprotected in OCT compound and sectioned coronally at 100-μm thickness using a cryostat. Sections were air-dried protected from light and then sequentially stained with solutions 4 and 5 as specified in the kit protocol. Following staining, sections underwent graded ethanol dehydration (70%, 95%, and 100%), xylene clearing, and permanent mounting. Neuronal morphology was analyzed in a blinded manner. Four to five neurons from the right prefrontal cortex were randomly sampled per rat (six rats/group). All slices were coded by an independent researcher, and measurements were performed without knowledge of group assignments before decoding for statistical analysis (20, 21). Dendritic morphology was assessed through Sholl analysis implemented as follows: complete 2D dendritic arbors were reconstructed; concentric spheres (10-μm radial increments) were projected from the soma centroid; and dendritic intersections with each sphere were counted to quantify branching complexity and spatial distribution. All morphometric analyses were conducted using the SNT plugin within the FIJI/ImageJ platform.

### 2.13 Tissue preparation and TEM imaging

Brain tissue samples were fixed in 2.5% glutaraldehyde for 4 h, followed by three washes with PBS. Subsequently, the samples were post-fixed in 1% osmium tetroxide for 2 h and rinsed again with PBS. After dehydration through a graded series of ethanol and acetone, the tissues were infiltrated with embedding resin at room temperature for 3 h. Following polymerization, ultrathin sections were prepared using an ultramicrotome and double-stained with uranyl acetate and lead citrate. The synaptic ultrastructure in the rat PFC was examined using a transmission electron microscope (TEM). Images were acquired at a magnification of ×20,000. Quantitative analysis, including active zone length, synaptic cleft width measurement, and postsynaptic density (PSD) thickness assessment, was performed using ImageJ software (27, 28).

### 2.14 Coimmunoprecipitation

Coimmunoprecipitation (Co-IP) assays were carried out following the manufacturer’s protocol for Protein A/G Magnetic Beads (HY-K0202, MedChemExpress, USA). The procedure began with washing the beads in binding/wash buffer, after which they were conjugated with specific antibodies—anti-ALK5 (Abcam, USA), anti-Gadd45b (Abcam, USA), or anti-IgG (Abcam, USA)—during a 2-h incubation at 4°C with constant rotation. Following removal of the supernatant and subsequent washing steps, protein samples were introduced and similarly rotated overnight at 4°C. After additional washes, the complexes were eluted using elution buffer, and the collected eluates were subjected to Western blot analysis. For Western blotting, the following antibodies were applied: anti-ALK5 (1:500, EPR20923-13, Abcam, USA), anti-Gadd45b (1:500, ab230646, Abcam, USA), HRP-Mouse Anti-Rabbit IgG Light Chain Specific (1:4,000, SA00001-7L,Proteintech, China), and horseradish peroxidase-conjugated goat anti-rabbit IgG (1:4,000, SA00001-2, Proteintech, China).

### 2.15 Enzyme-linked immunosorbent assay

The right PFC tissues from rats were rinsed with 1× PBS to eliminate residual blood, followed by homogenization in additional 1× PBS. The homogenates were kept at −20°C overnight. To disrupt cellular membranes, the samples underwent multiple freeze-thaw cycles. Thereafter, the homogenates were centrifuged at 5,000 ×g for 5 min at 4°C. The resulting supernatant was promptly collected for analysis. Concentrations of serotonin (5-HT)and dopamine (DA) in the supernatant were quantified using commercial assay kits (Cusabio Biotechnology, China).

### 2.15 Statistical analysis

Statistical analysis and graphing were performed using GraphPad Prism version 10.0.1. Data are presented as mean ± standard deviation (x ± s). Data collection was performed by a researcher who was unaware of the study’s specific details. We employed Student’s t-test, ANOVA (analysis of variance), and Tukey’s *post-hoc* test or two- way repeated measured ANOVA with a *post-hoc* Bonferroni test. p < 0.05 was considered statistically significant. Statistical methods for each experiment are detailed in the corresponding figure legend.

## 3 Results

### 3.1 Physiological parameters

All measured physiological parameters—including blood pressure, heart rate, and blood gases—remained within normal limits and showed no significant differences between groups (**Table 1**), consistent with our prior observations (14).

**Table 1:** Physiological parameters during the experiment (all data are shown as the mean ± SD).

Group	Mean blood pressure (mmHg)	Heart rate (bp/min)	pH	PCO_2_ (mmHg)	PO_2_ (mmHg)
Sham	83 ± 7.1	360 ± 10	7.36 ± 0.01	46.4 ± 0.8	116.1 ± 10.2
PSD	85 ± 8.2	365 ± 8	7.34 ± 0.03	44.7 ± 1.1	110 ± 11.3
PSD+ta-VNS	82 ± 9.4	370 ± 9	7.37 ± 0.02	45.5 ± 2.4	108.7 ± 12.5
PSD+ta-VNS+AAV-Scramble	84 ± 7.3	368 ± 11	7.35 ± 0.03	48.7 ± 3.1	109.5 ± 11.5
PSD+ta-VNS+AAV-shALK5	84 ± 9.1	362 ± 9	7.36 ± 0.02	44.2 ± 2.2	114.1 ± 13.4

### 3.2 The expression of the ALK5/Smad2/3/Gadd45β in the PFC following PSD

**Figure 2:**
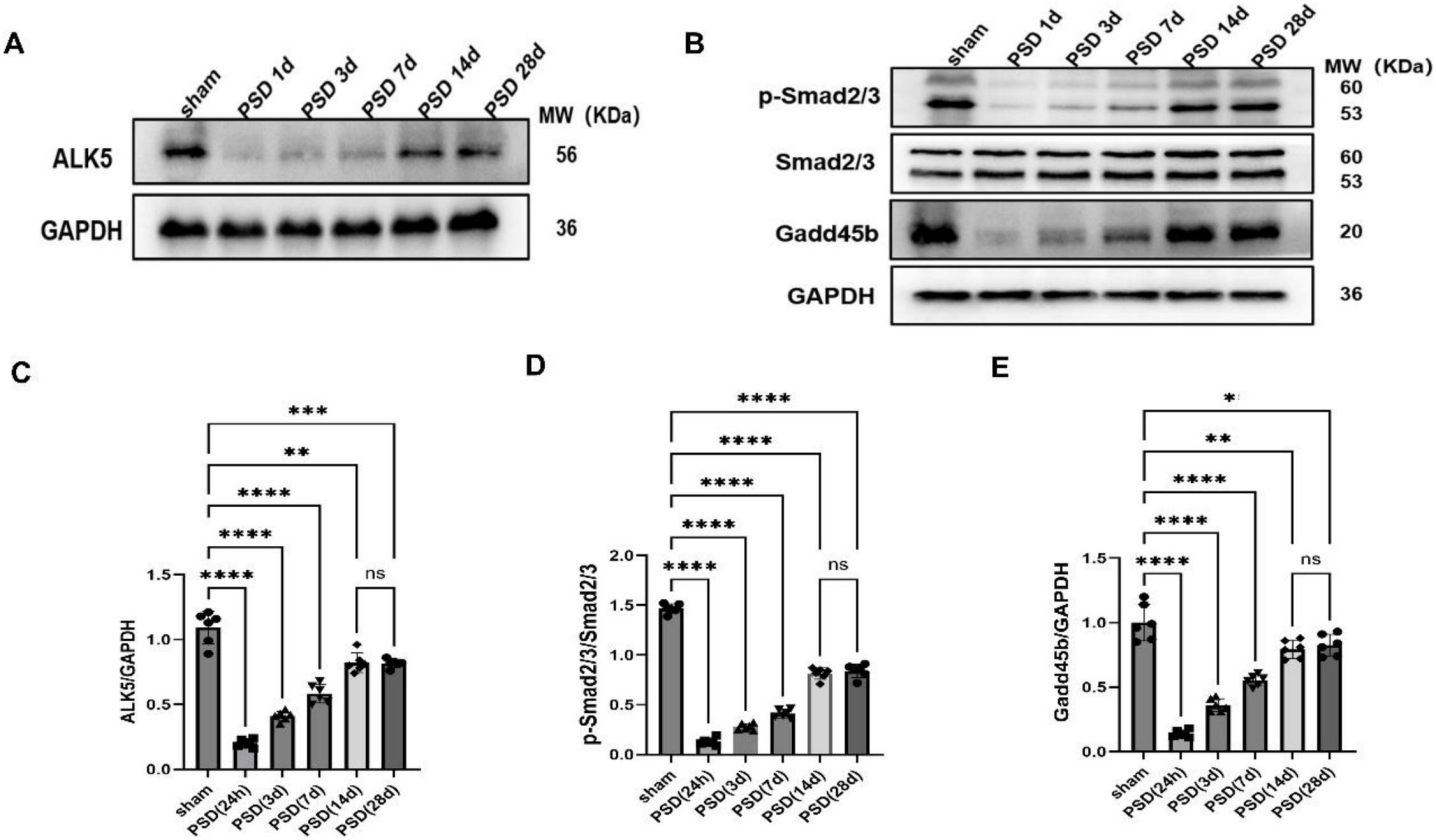
**(A–E)** Representative WB images and quantitative analysis of ALK5/Smad2/3/Gadd45β expression following PSD. The expression of ALK5, p-Smad2/3, and Gadd45β decreased in the PSD group as compared with that in the sham group on 1, 3, 7, 14, and 28 days. ALK5: Sham vs. PSD (24h)(t=16.89, df=10, p<0.0001); Sham vs. PSD (3d)(t=12.77,df=10,p<0.0001); Sham vs. PSD (7d)(t=8.65,df=10,p<0.0001); Sham vs. PSD (14d)(t=4.54,df=10,p<0.01); Sham vs. PSD (28d)(t=5.22,df=10,p<0.001). p-Smad2/3/Smad2/3: Sham vs. PSD (24h)(t=56.60,df=10,p<0.0001); Sham vs. PSD (3d)(t=50.80,df=10,p<0.0001); Sham vs. PSD (7d)(t=42.06,df=10,p<0.0001); Sham vs. PSD (14d)(t=21.59,df=10, p<0.0001); Sham vs. PSD (28d)(t=18.74,df=10, p<0.0001). Gadd45b: Sham vs. PSD (24h)(t=14.63,df=10,p<0.0001); Sham vs. PSD (3d)(t=10.43,df=10, p<0.0001);Sham vs. PSD (7d)(t=7.40,df=10,p<0.0001); Sham vs. PSD (14d)(t=3.20,df=10, p<0.01); Sham vs. PSD (28d)(t=2.60,df=10, p<0.05). *P < 0.05,**P < 0.01, ***P < 0.001, ****P < 0.0001, versus the sham group. n=6 rats/group/time point, unpaired two-tailed Student’s t-test. ns, not significant.

To investigate the time course of ALK5, phosphorylation of Smad2/3, and Gadd45β expression in the PFC of PSD rats, we performed WB analysis at 1, 3, 7, 14, and 28 days after PSD. Compared with the sham group, the PSD group showed a significant decline of ALK5 and Gadd45β, along with reduced Smad2/3 phosphorylation, at 1 day. Subsequently, the expression of the ALK5/Smad2/3/Gadd45β signaling axis proteins increased progressively and, by the final observation time point of 28 days, had failed to return to normal levels (**Figures 2A–E**). These findings suggested that PSD was associated with the downregulation of the ALK5/Smad2/3/Gadd45β signaling pathway.

### 3.3 ALK5 expression was enhanced by ta-VNS treatment following PSD

**Figure 3:**
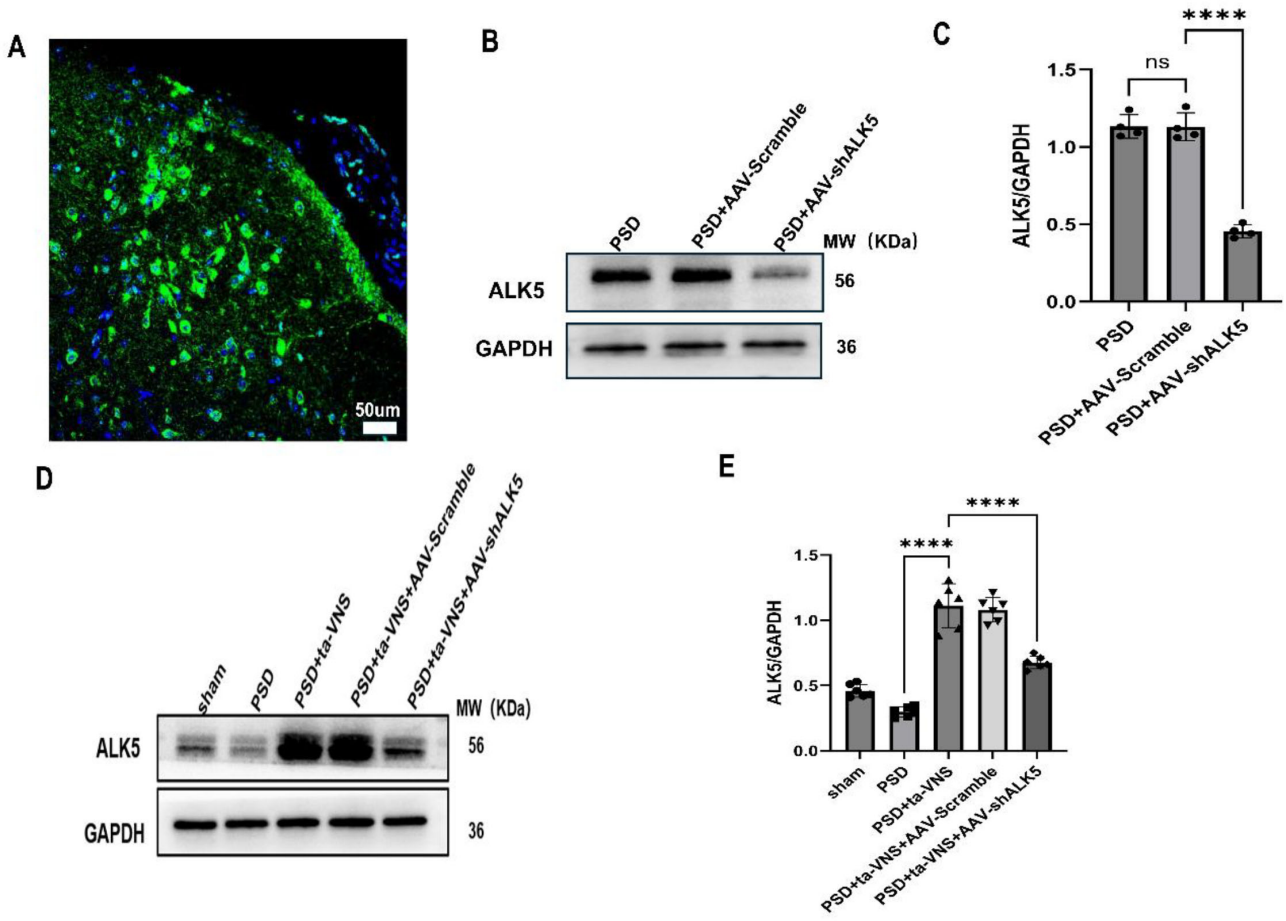
**(A)** Representative fluorescent images of EGFP (green) in the right PFC 39 days after AAV was stereotaxically injected. Scale bar = 50 μm. **(B, C)** WB images and quantitative analysis for ALK5 expression with AAV-shALK5 or AAV-Scramble injection at 1 day before MCAO/R (unpaired two-tailed Student’s t-test, n=4 rats/group). PSD+AVV-Scramble vs. PSD+AVV-shALK5 (t=13.57,df=6,p<0.0001) (D, E) WB images and quantitative analysis for ALK5 (n =6 rats/group). A significant upregulation of ALK5 was observed in the PSD+ta-VNS group compared with the PSD group following 14 days of ta-VNS treatment. Data are expressed as mean ± SD, ****P <0.0001. ANOVA followed by Tukey’s *post-hoc* test. The ANOVA revealed a significant effect of treatment, F (4, 25) = 91.91, P <0.0001. ns, not significant.

**Figure 4:**
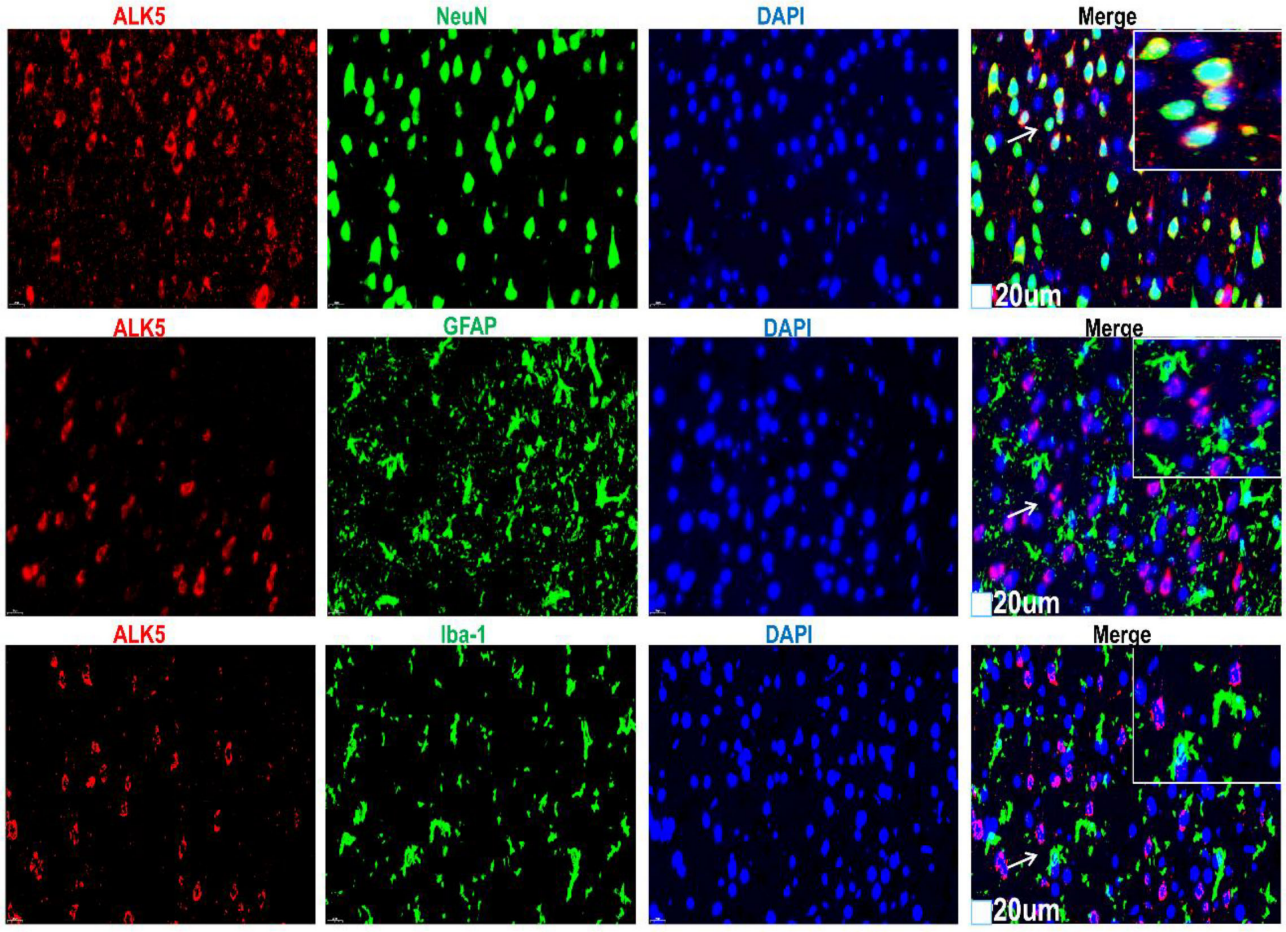
Representative images of IF staining for ALK5 (red), NeuN (green)/GFAP (green)/IBA1 (green), and cellular nuclei (blue). Scale bar = 20 μm. Arrows show positive cells, and the inserted images show magnified images of representative cells.

To investigate the possible relationship between the protective mechanisms of ta-VNS and ALK5 in a rat model of PSD, 1 day before establishing the rat model of MCAO/R, AAV-shALK5 was injected into the right PFC of the rats to knockdown the expression of ALK5 (**Figures 3A–C**). Furthermore, the expression and localization of ALK5 in the PFC were assessed using WB and IF staining. Since there was no statistically significant difference in ALK5 expression between the 14th and 28th days following PSD, we chose day 14 as the intervention time point. After 14 consecutive days of ta-VNS treatment, the PSD+ta-VNS group exhibited a marked upregulation of ALK5; however, this effect was abolished following ALK5 silencing (**Figures 3D, E**). In addition, ALK5 was present in both the cell membrane and cytoplasm, and it co-localized with the neuronal marker NeuN, but not with the astrocyte marker GFAP or the microglial marker Iba-1 (**Figure 4**).

### 3.4 Inhibition of ALK5 weakened the improvements in behavioral tests induced by ta-VNS in PSD rats

**Figure 5:**
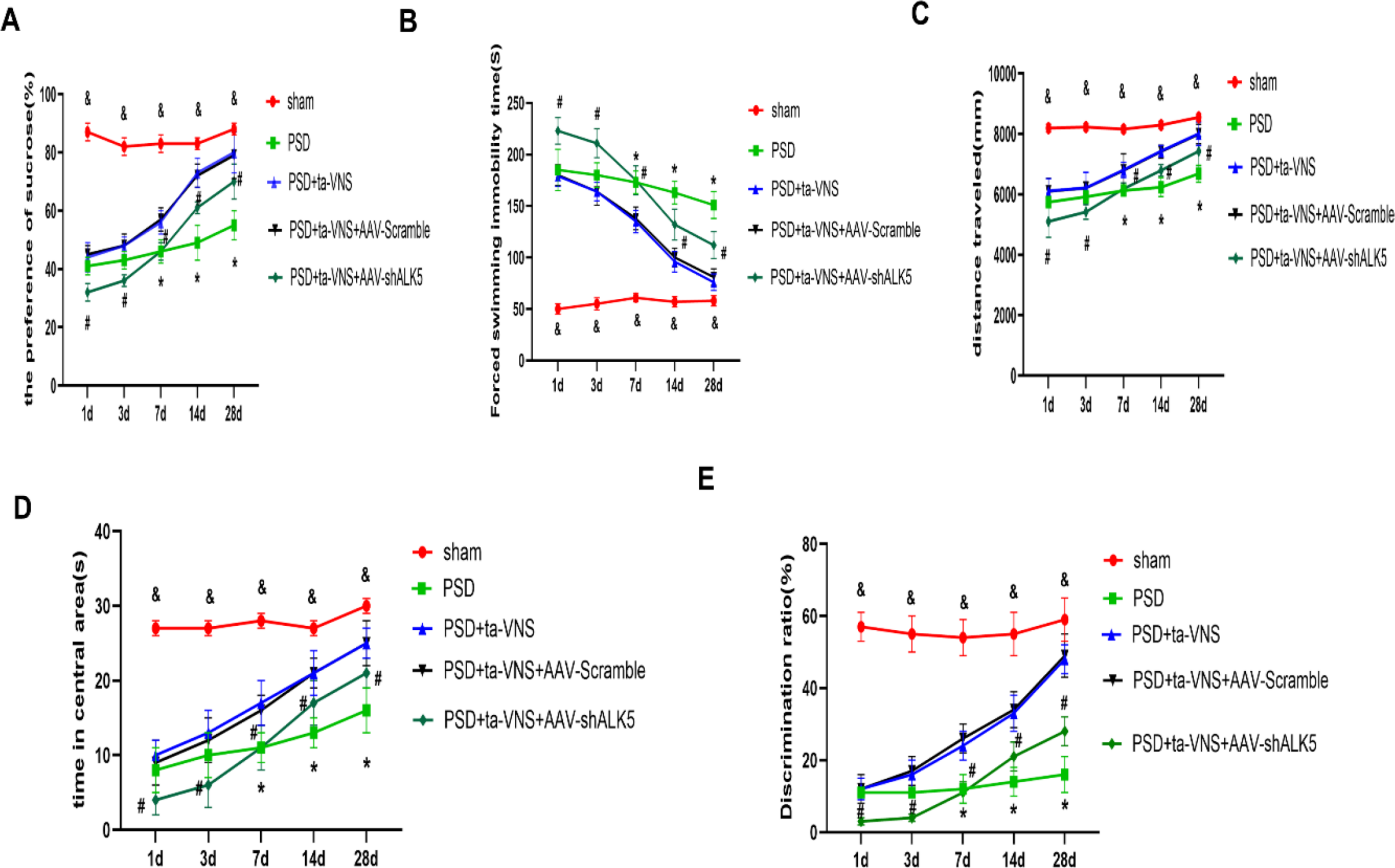
**(A)** SPT was used to estimate animal hedonic capacity by measuring sucrose solution intake. The two-way ANOVA revealed a significant time × therapy interaction (F (16, 125) = 17.72, P<0.0001). **(B)** FST was used to evaluate depression-like responses by exposing animals to an inescapable water tank. The two-way ANOVA revealed a significant time × therapy interaction F (16, 125) = 24.73, P<0.0001). **(C)** Distance traveled in the central area of the OFT. The two-way ANOVA revealed a significant time × therapy interaction (F (16, 125) = 8.40, P<0.0001). **(D)** Time spent in the central area of the OFT. The two-way ANOVA revealed a significant time × therapy interaction (F (16, 125) = 7.53, P<0.0001). **(E)** NOR test was performed to evaluate animal learning and memory capability. The two-way ANOVA revealed a significant time × therapy interaction (F (16, 125) = 15.64, P<0.0001). Sham vs. PSD, ^&^p < 0.0001; PSD vs. PSD+ta-VNS, *p < 0.01; PSD+ta-VNS vs. PSD+ta-VNS+AVV-shALK5, ^#^p < 0.05. n=6 rats/group, two-way repeated measures ANOVA followed by Bonferroni *post-hoc* test.

**Figure 6:**
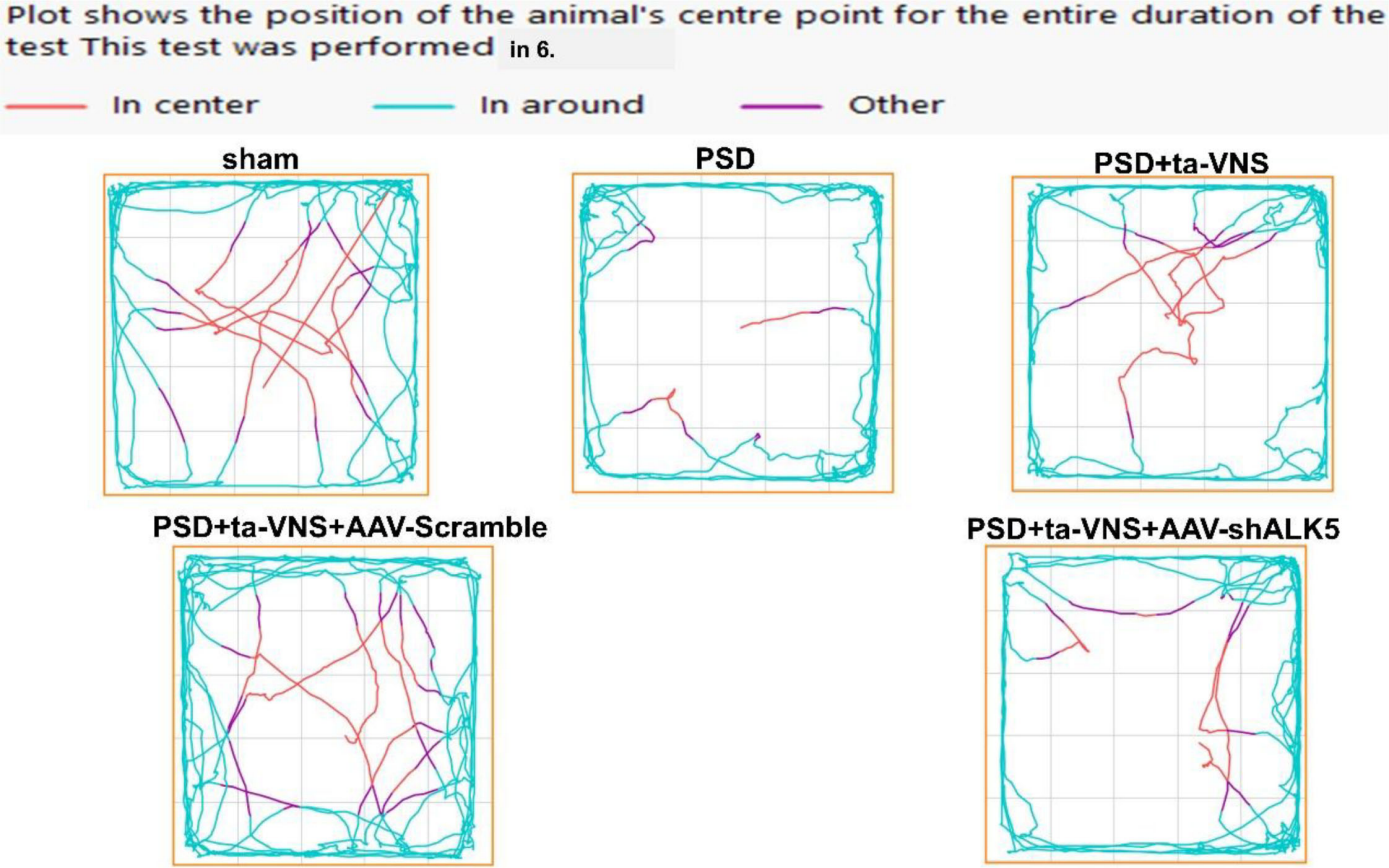
Representative tracks of the five groups in the OFT.

To evaluate the impact of ALK5 on depression-like behavior following PSD combined ta-VNS therapy, rats underwent a series of neurobehavioral assessments (SPT, FST, OFT, and NOR) conducted 1, 3, 7, 14, and 28 days after PSD. Rats in the PSD group showed a significant decrease in sucrose preference rate, distance traveled, time spent in the central area, and discrimination ratio, as well as a significant increase in immobility time compared with the sham group at every time point, confirming that PSD led to persistent depression-like behavior. As time went on, the depressive symptoms lessened, but by 28 days, they still had not returned to normal. In contrast, ta-VNS treatment reversed the above changes. Although ineffective in the initial phase (days 1 and 3), a significant antidepressant effect emerged after 7 days of treatment (at the day 7 and day 14 assessments) compared with the PSD group, and this benefit was maintained at day 28, after the 14-day treatment course had ended. Interestingly, when AAV-shALK5 was co-administered, the positive effects of ta-VNS on depression-like behavior were substantially diminished at the same time points (**Figures 5A–E**, **6**). These findings demonstrated that inhibition of ALK5 signaling compromises the efficacy of ta-VNS in attenuating depressive-like behaviors post PSD. This highlighted the critical role of the ALK5 pathway in mediating the therapeutic effects of ta-VNS.

### 3.5 Inhibition of ALK5 attenuated the ta-VNS-mediated suppression of neuronal damage of PFC in PSD rats and diminishes its enhancing effect on monoamine neurotransmitters

**Figure 7:**
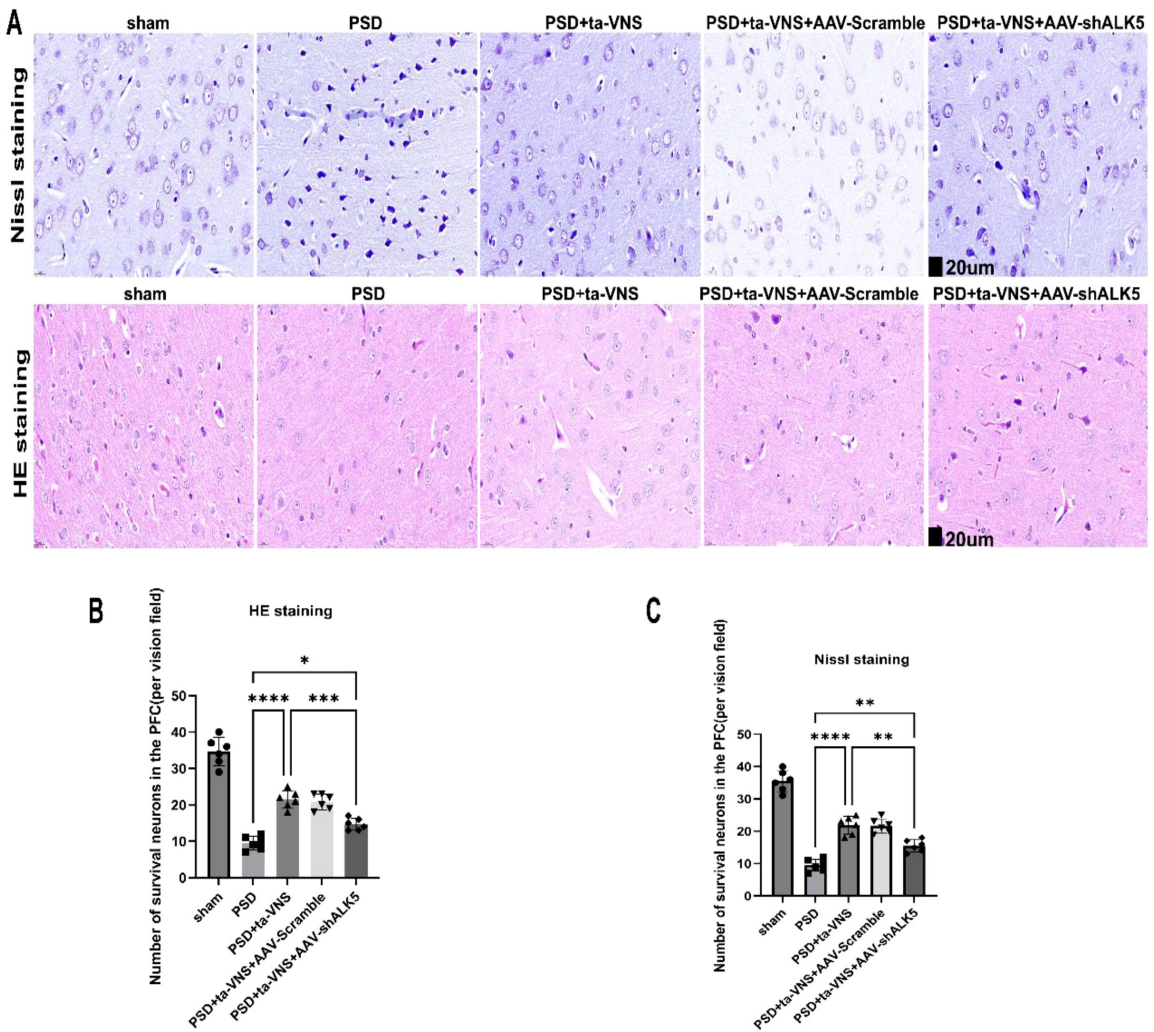
Impact of ta-VNS on neurons within the PFC. **(A)** Representative images of Nissl staining and HE staining results in each group (scale bar =  20  μm). **(B)** The number of survival neurons using HE staining was represented in the bar graph. ANOVA revealed a significant effect of treatment, F(4, 25) = 84.34, P <0.0001. **(C)** The number of survival neurons using Nissl staining was represented in the bar graph. ANOVA revealed a significant effect of treatment, F (4, 25) = 93.01, P <0.0001. Data are expressed as mean ± SD, *p < 0.05, **P < 0.01, ***P <0.001, ****P <0.0001. n=6 rats/group, ANOVA followed by Tukey’s *post-hoc* test.

To further investigate the role of ALK5 in the neuroprotective effects of ta-VNS in PSD rats, we assessed Nissl staining, HE staining, and TUNEL staining in rats 14 days after inducing PSD. As shown in **Figures 7A, B**, neurons in the sham group exhibited normal cellular morphology. In contrast, PSD resulted in significant neuronal damage in the PFC, characterized by vacuolization, disorganized structure, reduced Nissl bodies, denaturation, and necrosis. Compared with the PSD group, ta-VNS treatment appeared to reduce neuronal damage, showing a higher density of surviving neurons and relatively intact cellular structures. However, this neuroprotective effect was abolished in the PSD+ta-VNS+AAV-shALK5 group compared with the PSD+ta-VNS+AAV-Scramble group. The pathological changes observed through Nissl staining in the PFC were consistent with those seen in HE staining (**Figures 7A, B**).

**Figure 8:**
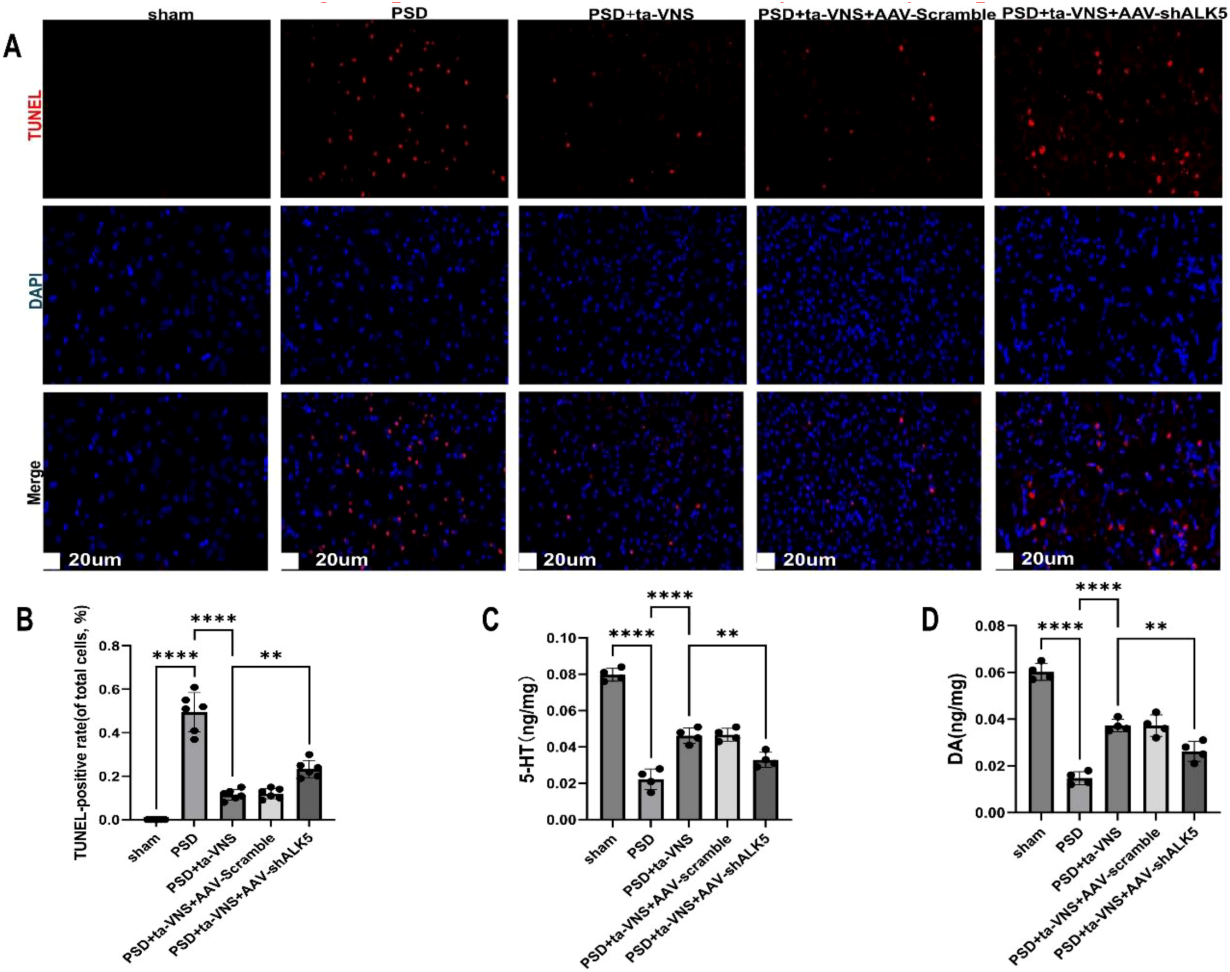
Impact of ta-VNS on apoptosis within the right PFC. **(A)** Representative fluorescent images of TUNEL-labeled cells of each group (scale bar = 20  μm). **(B)** TUNEL-positive rates was represented in the bar graph (n = 6 rats/group). The ANOVA revealed a significant effect of treatment, F (4, 25) = 98.93, P <0.0001. **(C)** Changes of neurotransmitter levels 5-HT in the right PFC of PSD rats treated with ta-VNS and AAV-shALK5 (n=4 rats/group). The ANOVA revealed a significant effect of treatment, F (4, 15) = 102.5, P <0.0001. **(D)** Changes of neurotransmitter levels DA in the right PFC of PSD rats (n=4 rats/group). The ANOVA revealed a significant effect of treatment, F (4, 15) = 85.17, P <0.0001. Data are expressed as mean ± SD, **P <0.01, ****P <0.0001. ANOVA followed by Tukey’s *post-hoc* test.

Given that apoptosis often follows PSD, we used TUNEL staining to quantify apoptosis in the PFC across groups. As shown in **Figure 8**, virtually no apoptotic cell was observed in the sham group. Conversely, the PSD group exhibited a significant increase in apoptosis. ta-VNS treatment significantly reduced the number of apoptotic cells compared with the PSD group, whereas ALK5 silencing partially attenuated this effect (**Figures 8A, B**). These results indicated that ta-VNS treatment ameliorated cell damage and death in the PFC of PSD through the ALK5 signaling pathway.

Additionally, we measured the levels of monoamine neurotransmitters in the PFC (**Figures 8C, D**). The PSD group showed lower 5-HT and DA levels at 14 days following PSD than the sham group. The administration of ta-VNS significantly elevated 5-HT and DA levels. However, this increase was blocked by AAV-shALK5. These findings suggest that ta-VNS helped increase monoamine neurotransmitter levels in PSD model rats.

### 3.6 Inhibition of ALK5 attenuated the ta-VNS-mediated neurogenesis in the PFC after inducing PSD

**Figure 9:**
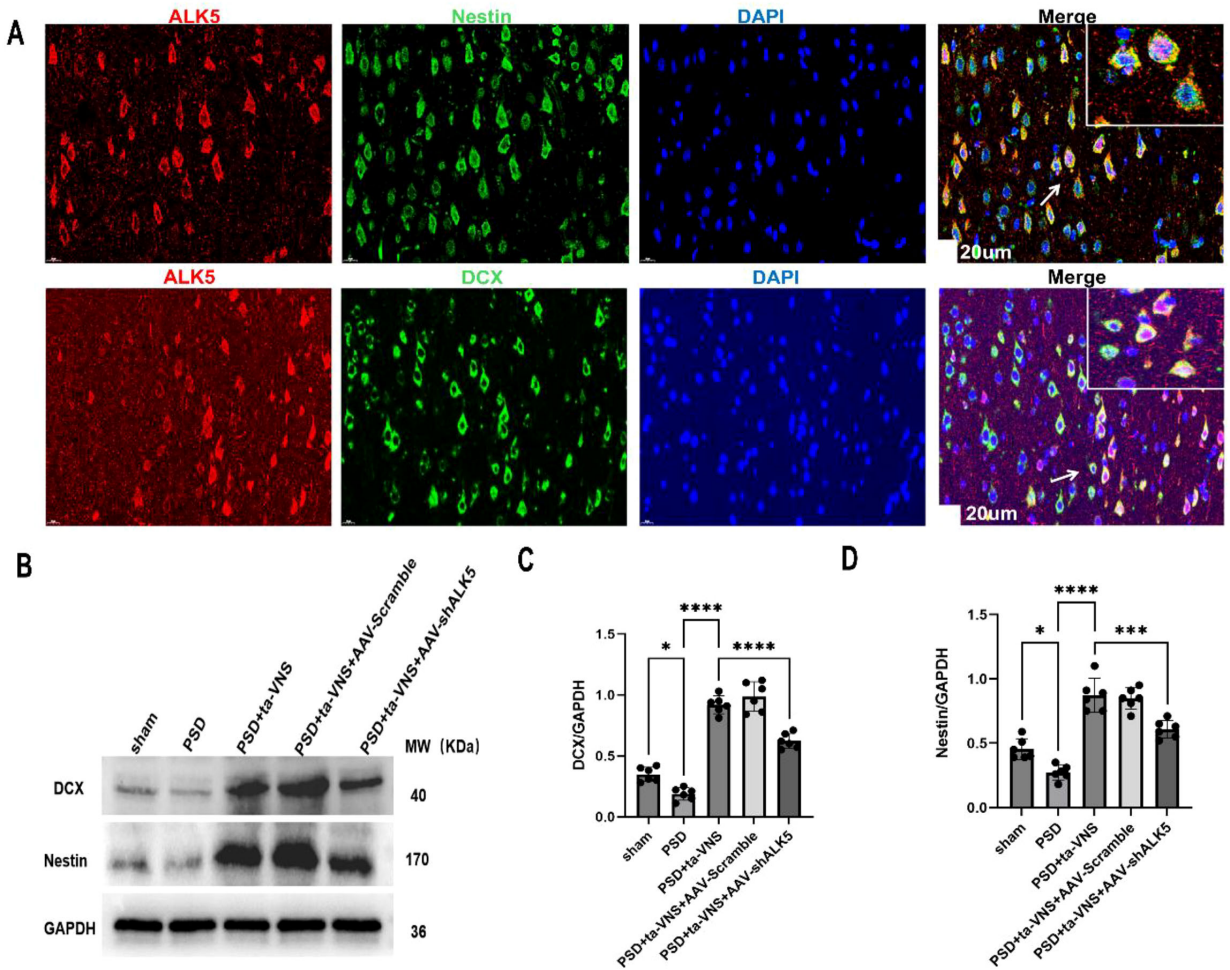
Neurogenesis was promoted by ta-VNS treatment via regulating the ALK5 signaling pathway. **(A)** Representative images of IF staining for ALK5 (red), Nestin (green)/DCX (green), and cellular nuclei (blue) (scale bar = 20 μm). Arrows show the positive cells, and the inserted images show magnified images of representative cells. **(B)** Representative images of DCX and Nestin expression. **(C)** Quantitative analysis of DCX expression. ANOVA revealed a significant effect of treatment, F (4, 25) = 121.1, P <0.0001. **(D)** Quantitative analysis of Nestin expression. ANOVA revealed a significant effect of treatment, F (4, 25) = 51.32, P <0.0001. Data are expressed as mean ± SD, *P <0.05,***P <0.001, ****P <0.0001. n=6 rats/group, ANOVA followed by Tukey’s *post-hoc* test.

To investigate the role of ALK5 in neurogenesis after inducing PSD, we performed double IF labeling to assess its relationship with Nestin and DCX. After PSD, ALK5 was found to colocalize with Nestin (a neural stem cell marker) and DCX (a marker for neuroblasts and immature neurons) (**Figure 9A**). This finding suggested that ALK5 may play a key role in neurogenesis in PSD rats. In line with the IF results, WB analysis also demonstrated that ta-VNS treatment upregulated the expression of DCX and Nestin proteins. However, this upregulation was attenuated by the administration of AAV-shALK5 (**Figures 9B–D**). These results suggested that ta-VNS treatment promoted neurogenesis, at least in part, by modulating the ALK5 signaling pathway.

### 3.7 Ta-VNS treatment mediated axonal regeneration and reorganization by ALK5 signaling pathway

**Figure 10:**
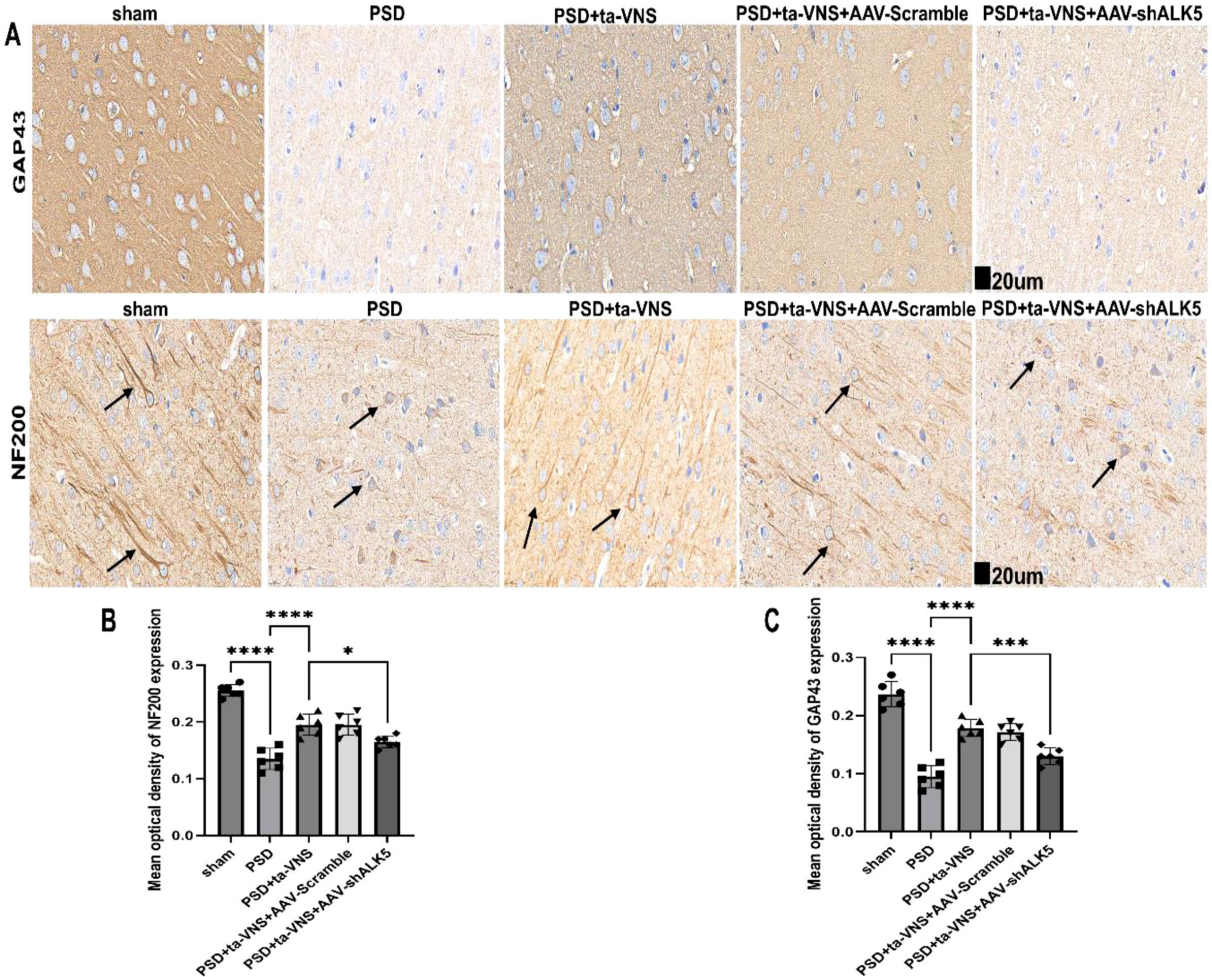
Ta-VNS treatment mediated axonal regeneration and reorganization by the ALK5 signaling pathway. **(A, B)** IHC staining images and analysis of NF–200 expression. Arrows show positive fibers (scale bar=20μm). ANOVA revealed a significant effect of treatment, F (4, 25) = 46.77, P <0.0001. **(A, C)** IHC staining images and analysis of GAP43 expression. Arrows show positive fibers (scale bar = 20μm). ANOVA revealed a significant effect of treatment, F (4, 25) = 59.21, P <0.0001. Data are expressed as mean ± SD, *P <0.05, ***P <0.001, ****P <0.0001. n=6 rats/group, ANOVA followed by Tukey’s *post-hoc* test.

Axonal regeneration was evaluated by assessment of NF-200 and GAP-43. The IHC staining of NF-200 (**Figures 10A, B**) showed intact nerve fibers in the sham group. In contrast, the PSD group exhibited severe nerve fiber damage following a 14-day period after PSD induction, characterized by a marked reduction in fiber density and disorganized arrangement. Treatment with ta-VNS in the PSD + ta-VNS group led to an increase in nerve fiber quantity and a more organized structure. However, in the PSD+ta-VNS+AAV-shALK5 group, ALK5 silencing markedly attenuated the restorative effect of ta-VNS, resulting in a significant reduction in nerve fiber number. The results of immunohistochemistry of GAP43 were similar to those of NF-200 (**Figures 10A, C**). The results showed that ALK5 is critical for the axonal regeneration and reorganization promoted by ta-VNS treatment.

### 3.8 Ta-VNS treatment promoted dendritic plasticity by modulating the ALK5 signaling pathway

**Figure 11:**
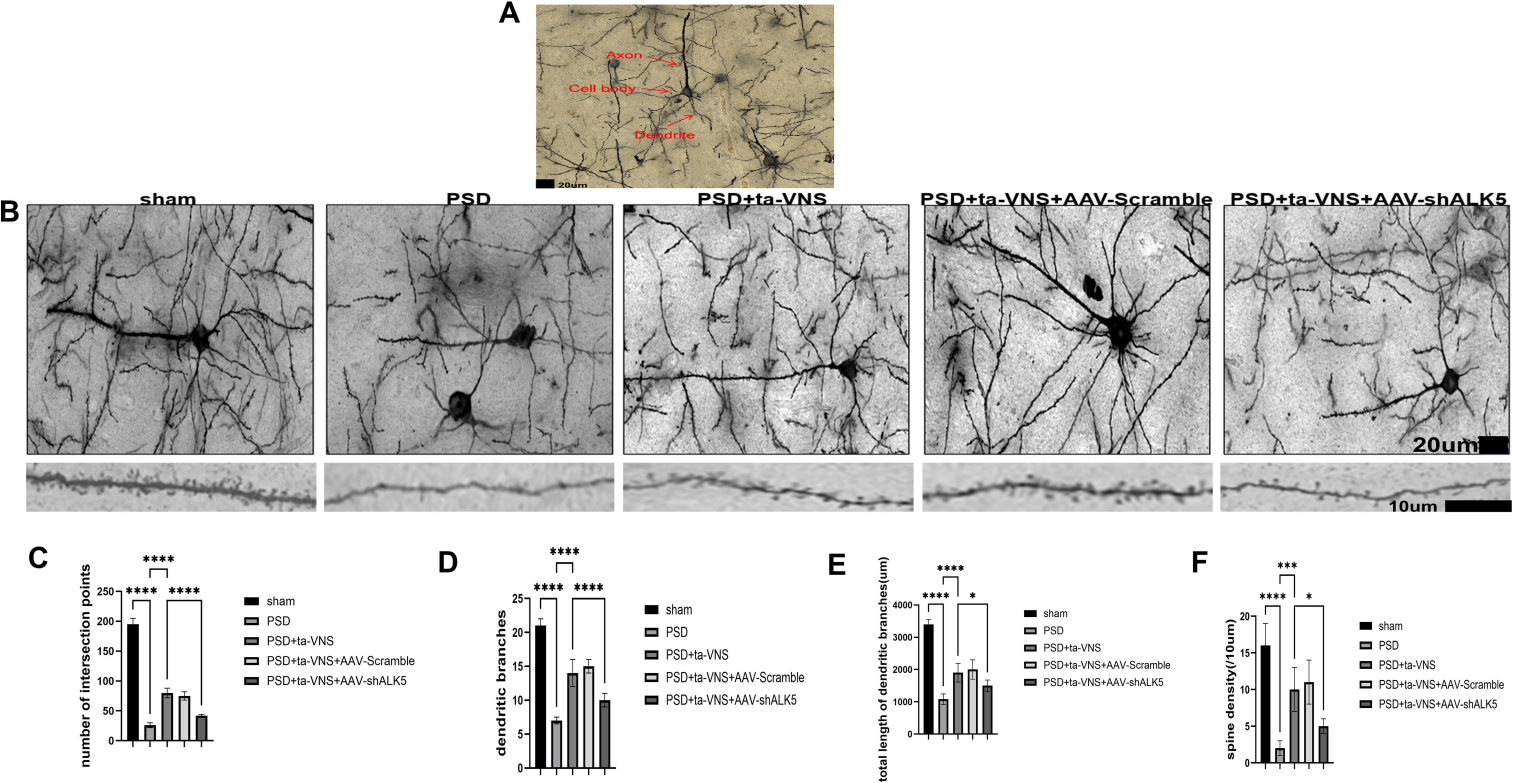
Ta-VNS treatment modulated dendritic plasticity by targeting the ALK5 signaling pathway. **(A)** Representative Golgi-Cox staining section. Scale bars: 20 μm in **(A, B)** Representative images of Golgi-Cox-stained pyramidal neurons after inducing PSD. Scale bar = 20 μm. **(C)** Comparison of the number of intersection points in Sholl analysis. ANOVA revealed a significant effect of treatment, F (4, 25) = 564.8, P <0.0001. **(D)** Number of dendritic branches. ANOVA revealed a significant effect of treatment, F (4, 25) = 117.1, P <0.0001. **(E)** Total dendritic length quantification. ANOVA revealed a significant effect of treatment, F (4, 25) = 90.29, P <0.0001. **(F)** Quantitation of the dendritic spine density. ANOVA revealed a significant effect of treatment, F (4, 25) = 30.72, P <0.0001. Data are expressed as mean ± SD, *P <0.1, ****P <0.0001. n=6 rats/group, ANOVA followed by Tukey’s *post-hoc* test.

**Figure 12:**
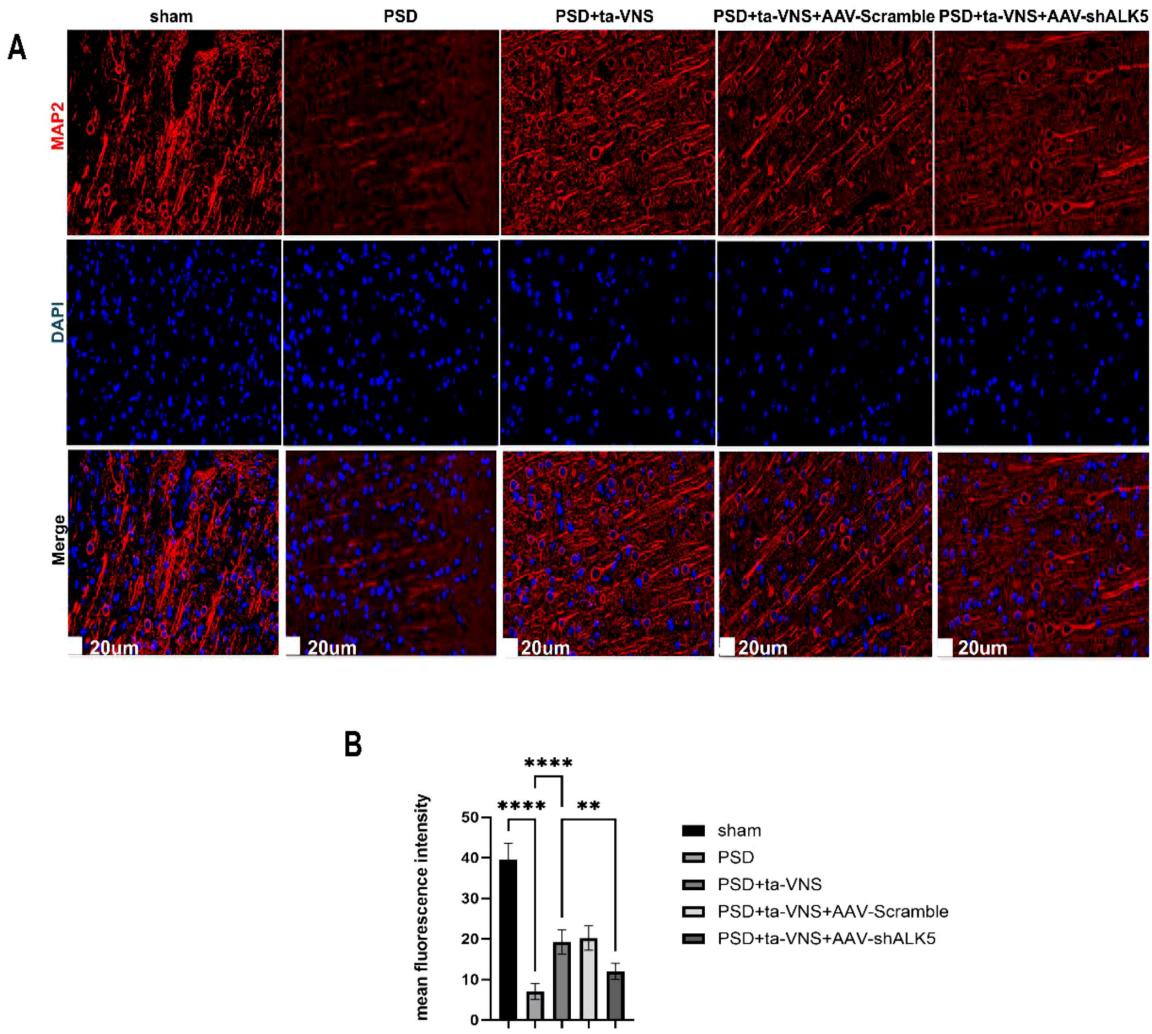
Ta-VNS treatment modulated dendritic plasticity by targeting the ALK5 signaling pathway. **(A)** Representative images of IF staining for MAP2 (red) and cellular nuclei (blue) (scale bar = 20 μm). **(B)** Mean fluorescence intensity in the bar graph. ANOVA revealed a significant effect of treatment, F (4, 20) = 91.82, P <0.0001. Data are expressed as mean ± SD, **P <0.01, ****P <0.0001. n=5 rats/group, ANOVA followed by Tukey’s *post-hoc* test.

Dendritic morphology was analyzed in Golgi-Cox-stained sections (**Figure 11A**). Following 2D reconstruction of neuronal dendrites, Sholl analysis was employed to quantify dendritic complexity by measuring the number of intersections between dendrites and equidistant concentric circles. As shown in **Figures 11B–E**, the sham group exhibited the highest number of Sholl intersections. PSD injury significantly reduced dendritic complexity, resulting in the lowest number of intersections in the PSD group. Although ta-VNS treatment (PSD+ta-VNS group) increased the number of intersections, this beneficial effect was abolished by AAV-shALK5 treatment. Additionally, we measured the number and total length of dendritic branches and dendritic spines, which revealed statistically significant differences. The fluorescence expression trend of the dendritic marker MAP2 was consistent with the aforementioned findings (**Figures 12A, B**). These results indicated that ta-VNS treatment enhanced dendritic plasticity by promoting the growth in the number and length of dendritic branches and the number of dendritic spines. This effect was mediated through the ALK5 signaling pathway.

### 3.9 Ta-VNS treatment improved synaptic ultrastructure and upregulated the expression of synapse‐associated proteins through the ALK5 signaling pathway

**Figure 13:**
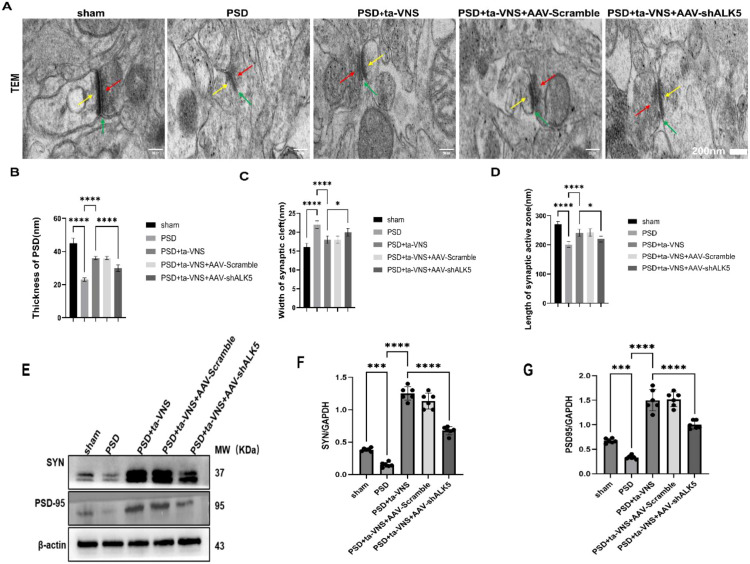
**(A)** Representative images of TEM for each experimental group. Scale bar = 200 nm. **(B)** Image analysis of PSD thickness. ANOVA revealed a significant effect of treatment, F (4, 25) = 124.7, P <0.0001. **(C)** Image analysis of the width of the synaptic cleft. ANOVA revealed a significant effect of treatment, F (4, 25) = 31.20, P <0.0001. **(D)** Image analysis of the length of the synaptic active zone. ANOVA revealed a significant effect of treatment, F (4, 25) = 32.92, P <0.0001. **(E)** Representative images of WB images of PSD-95 and SYN expression (n = 6 biological replicates). **(F)** Quantitative analysis of SYN expression. ANOVA revealed a significant effect of treatment, F (4, 25) = 211.1, P <0.0001. **(G)** Quantitative analysis of PSD95 expression. ANOVA revealed a significant effect of treatment, F (4, 25) = 100.1, P <0.0001. Data are expressed as mean ± SD, *P <0.05, ***P <0.001,****P <0.0001. n=6 rats/group, ANOVA followed by Tukey’s *post-hoc* test. PSD, postsynaptic density. Yellow arrows show thickness of PSD; red arrows show active zone length; green arrows show width of synaptic cleft.

To further evaluate synaptic plasticity, synaptic ultrastructure was examined via TEM. Representative images are presented in **Figure 13A**. Examination of synapses in the PSD rat model revealed clear ultrastructural damage, with features including fewer synaptic vesicles, blurred and faint vesicles, blurred presynaptic membrane, thinned presynaptic dense material, a reduced active zone length, and a widened synaptic cleft (**Figures 13B–D**). These changes are detrimental to synaptic signal transmission. Although ta-VNS ameliorated the aforementioned synaptic ultrastructural abnormalities,AAV-shALK5 administration abolished this beneficial effect. Synapse‐associated proteins, including SYN and PSD-95, play crucial roles in plasticity. In this study, we utilized WB analysis to assess the expression levels of synapse–associated proteins in the PFC. As depicted in **Figures 13E–G**, in the PSD group, compared with the sham group, the WB analysis revealed a significant reduction in the levels of PSD-95 and SYN expression. However, treatment with ta-VNS led to a notable increase in the levels of SYN and PSD‐95, whereas this effect was counteracted by AAV-shALK5. This indicated that ta-VNS treatment restored the synaptic ultrastructure and increased the expression of synapse‐associated proteins of PSD model rats, which helped to mitigate depression-like behaviors.

### 3.10 Inhibition of ALK5 attenuated the ta-VNS-induced increase in the expression of Smad2/3/Gadd45β expression after inducing PSD

**Figure 14:**
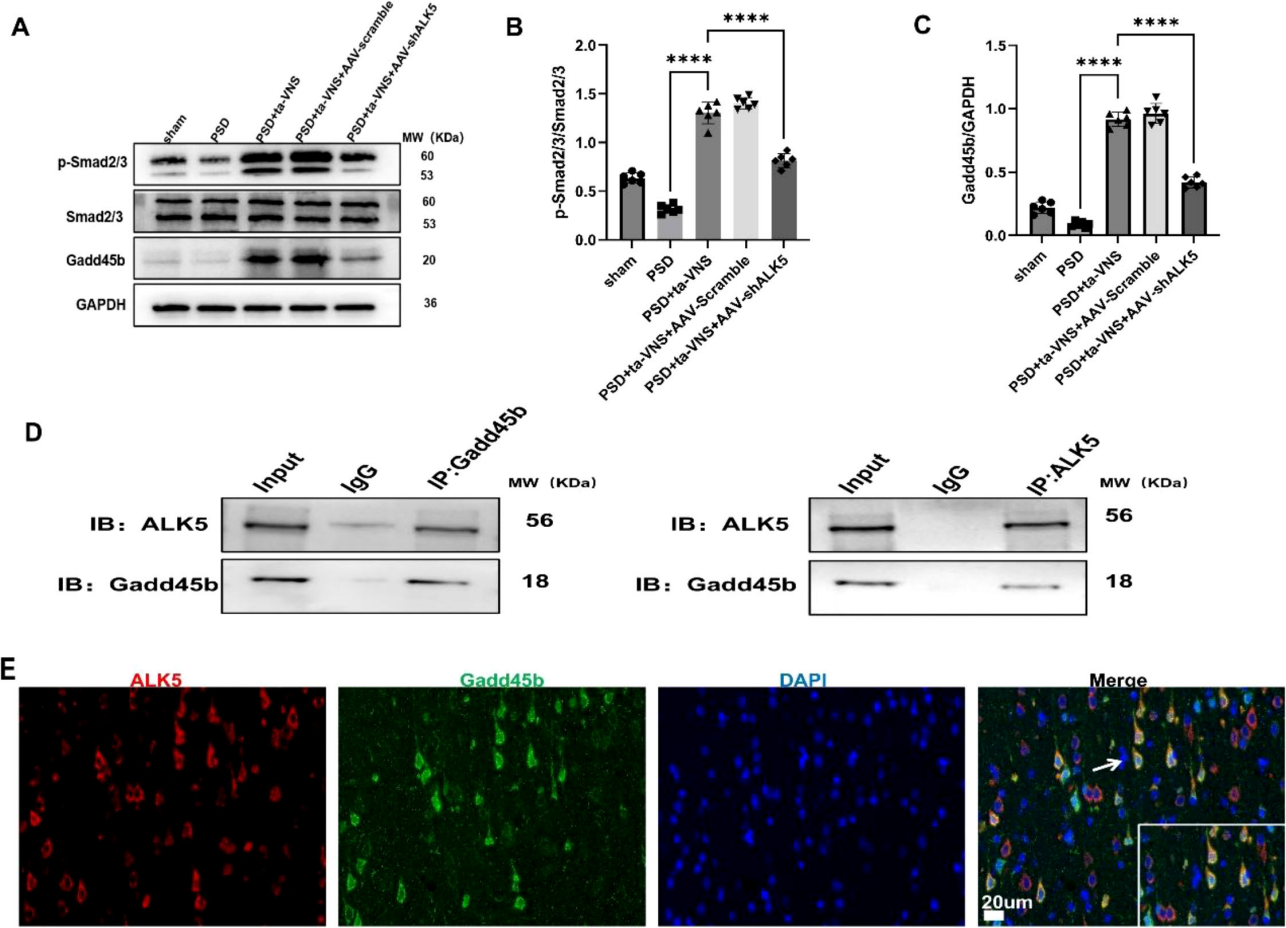
ta-VNS treatment upregulated ALK5 to promote the phosphorylation of Smad2/3 and Gadd45β expression. **(A)** Representative images of WB images of Smad2/3/Gadd45β expression. **(B)** Quantitative analysis for Smad2/3 expression. ANOVA revealed a significant effect of treatment, F (4, 25) = 245.5, P <0.0001. **(C)** Quantitative analysis for Gadd45β expression. ANOVA revealed a significant effect of treatment, F (4, 25) = 346.2, P <0.0001. The expression of p-Smad2/3 and Gadd45β increased in the PSD+ta-VNS group as compared with that in the PSD group on 14 days after ta-VNS treatment. Data are expressed as mean ± SD, ****P <0.0001. n=6 rats/group, ANOVA followed by Tukey’s *post-hoc* test. **(D)** Coimmunoprecipitation experiments of ALK5 and Gadd45b in the PFC 14 days following PSD. **(E)** Representative images of IF staining for ALK5 (red), Gadd45b (green), and cellular nuclei (blue) SS(scale bar = 20 μm). Arrows show the positive cells, and the inserted images show magnified images of representative cells.

In the canonical TGF-β pathway, ALK5 promotes Smad2/3 phosphorylation, leading to the formation of a Smad2/3-Smad4 complex that translocates into the nucleus (29, 30), given evidence that the ALK5/Smad2/3 axis may upregulate Gadd45b (17). One day prior to inducing the MCAO/R model, AAV-shALK5 or AAV-Scramble was injected into the rat PFC. To investigate the impact of ta-VNS on the expression of Smad2/3, phospho-Smad2/3, and Gadd45β following PSD, we employed WB. WB analysis revealed that, compared with the PSD group, the PSD+ta-VNS group exhibited significant upregulation of the Smad2/3 phosphorylation ratio, and Gadd45β expression at 14 days post-ta-VNS treatment. Furthermore, the regulatory effect of ta-VNS on the Smad2/3/Gadd45β pathway was abolished upon ALK5 silencing (**Figures 14A–C**). In addition, co-immunoprecipitation assays confirmed an interaction between ALK5 and Gadd45b in the PFC at 14 days after inducing PSD (**Figure 14D**). Moreover, ALK5 was found to colocalize with Gadd45b in the PFC at 14 days after inducing PSD (**Figure 14E**). Collectively, these data indicated that ALK5 mediated Gadd45b protein expression by regulating Smad2/3 phosphorylation. Moreover, the ALK5/Smad2/3/Gadd45β signaling pathway was involved in the neuroplastic changes that ta-VNS regulated for treating PSD.

## 4 Discussion

PSD can severely impede patient recovery by diminishing cognitive function and autonomy, increasing suicide risk, and creating a vicious cycle (31). While pharmacotherapy, particularly SSRIs like fluoxetine and sertraline, remains the first-line treatment for core PSD symptoms (32), a substantial proportion of patients exhibit an inadequate antidepressant response (33). This limited efficacy is likely attributable to an incomplete understanding of the underlying neurobiological mechanisms of PSD. Emerging non-pharmacological therapies, such as electroconvulsive therapy and neuromodulation, have shown significant potential. These alternative approaches, which are based on the principles of neural circuit regulation, may enhance existing strategies for treating depression.

Ta-VNS is appealing as it is non-invasive and portable and can be self-administered in the home. For those reasons, there are early studies exploring the effects of ta-VNS on depression, with modest effect sizes reported (34–36). Accumulating clinical evidence has indicated its antidepressant action (37–39). Our previous clinical study (12) demonstrated that adding ta-VNS to standard pharmacological treatment significantly improves neuropsychiatric symptoms in PSD patients compared with standard treatment alone. Moreover, ta-VNS therapy was well-tolerated, with mild adverse effects primarily limited to minor local allergic skin reactions at the auricular site. These findings suggest that ta-VNS may be an effective and safe therapeutic approach for stroke patients. However, the potential molecular biological mechanisms underlying the anti-PSD effects of ta-VNS have not yet been investigated. This study investigates the therapeutic potential and underlying mechanism of ta-VNS in a rat model of PSD. Our study suggested that early ta-VNS intervention alleviated depressive-like behaviors and promoted neuroplasticity. The PFC is among the most severely affected regions in the brains of individuals with depressive disorder (40). Structural and functional abnormalities in the PFC are closely linked to depression-like behaviors, including negative processing bias, anhedonia, and learned helplessness (41). A meta-analysis on repetitive transcranial magnetic stimulation for PSD treatment has demonstrated that targeting the PFC produces significant antidepressant effects (42). Additionally, animal studies have further demonstrated that intranasal drug delivery exerts antidepressant effects in PSD rats. This effect is primarily mediated by the upregulation of BDNF expression in the PFC, with no significant changes observed in the hippocampus (43). Based on these findings, we focused on the PFC as the specific brain region for our study on PSD.

Our previous animal studies indicated that the ALK5 signaling pathway may be involved in neurological functional recovery after stroke (20, 21). In this study, ALK5 is constitutively expressed in the PFC region of brain tissue in the sham group. Its expression was found to decrease significantly on the first day after PSD, followed by a gradual increase over time. However, even at 28 days after PSD modeling, ALK5 expression had not returned to baseline levels. The downstream molecules of ALK5, namely, the phosphorylation of Smad2/3 and the expression changes of Gadd45β, are consistent with the direction of ALK5 changes. A positive correlation was observed between elevated ALK5/Smad2/3/Gadd45β signaling pathway and the amelioration of depressive symptoms in PSD rats. In addition, ALK5 was observed in the cytomembrane and cytoplasm and colocalized with the neuron NeuN. After 14 days of ta-VNS treatment, the expression of ALK5 was significantly increased, accompanied by an improvement in depressive symptoms. These results suggested that ALK5 upregulation may contribute to neuroprotection and recovery from depressive symptoms following PSD. In addition, our findings indicated that ta-VNS exerted a time-dependent therapeutic effect on depression-like symptoms. Although no early improvement was observed within the first 3 days of treatment, a significant amelioration in depression-like behaviors was noted after 7 days of ta-VNS treatment. Importantly, this therapeutic effect was sustained, as demonstrated by a 28-day follow-up at the end of the 14-day treatment, during which the ta-VNS + PSD group exhibited significantly lower levels of depression-like behaviors compared with the PSD group, whereas ALK5 silencing significantly abrogated the depressive-like behavior recovery promoted by ta-VNS following PSD, as evidenced by the impaired performance of the PSD+ta-VNS+AAV-shALK5 group compared with the PSD+ta-VNS group. These results indicated that ALK5 is involved in the improvement of depressive symptoms by ta-VNS treatment. Moreover, we employed HE and Nissl staining to examine the PFC structure in PSD rats. The results indicated that PSD modeling induced severe brain tissue edema, a loosened nerve fiber network, widespread neuronal degeneration and necrosis, and a reduction in Nissl bodies. Notably, following ta-VNS treatment, a significant improvement in the morphological structure of the PFC was observed, whereas ALK5 silencing partially attenuated this effect. In addition, ta-VNS significantly reduced the increased PFC apoptosis induced by PSD and increased the expression of emotion-associated monoamine neurotransmitters, 5-HT and DA. However, the neuroprotective effects of ta-VNS and its upregulation of neurotransmitters disappeared as ALK5 expression was downregulated. These findings further indicate that ALK5 may serve as a potential therapeutic target for the neuroimmune modulatory effects of ta-VNS, thereby contributing to the alleviation of post-stroke depression-like symptoms. However, the underlying mechanism remains to be fully elucidated.

Neuroplasticity is defined as the brain’s ability to undergo neurobiological changes in response to extrinsic stimuli such as chronic exposure to stress. It encompasses the processes of neural network growth and reorganization, which drives the restoration of neuropsychiatric function and serves as the fundamental basis for recovery from PSD (44–46). At the cellular level, neuroplasticity is reflected in the structural remodeling of neurons—including changes in dendritic branching and axonal sprouts—and quantitative shifts in dendritic spine, synaptic remodeling, and receptor density (47). Amelioration of depressive-like behavior based on neuroplasticity plays an essential role in functional recovery following PSD. Our previous study (20, 21) demonstrated that the upregulation of the ALK5 signaling pathway promotes post-stroke neuroplasticity, thereby improving neurological function. However, it remains unclear whether ALK5 can improve neuroplasticity in PSD. We further demonstrated that ta-VNS upregulated ALK5 to improve depressive-like behavior by showing that it promoted neurogenesis, as evidenced by increased levels of the newborn neuron markers DCX and Nestin. Nevertheless, the promotion of neurogenesis by ta-VNS was counteracted by silencing ALK5 expression. These results provided initial evidence that ta-VNS may regulate neuroplasticity through the ALK5 pathway.

In order to further confirm whether ALK5 improve depressive-like behaviors by affecting neuroplasticity induced by ta-VNS following PSD. Therefore, exploring methods to effectively measure neuroplasticity in PSD is essential. Toward this goal, Fan and Liu et al. (21, 48) employed Golgi-Cox staining to evaluate dendritic plasticity and used TEM to examine synaptic structure. Furthermore, NF-200, GAP-43, MAP-2, PSD-95, and SYN which are components of the neuronal cytoskeleton, serve as markers for axonal and dendrite regeneration and synaptic plasticity. Accordingly, this study utilized these methods to evaluate neuroplasticity. Our results showed that ta-VNS treatment promoted axonal and dendritic regeneration and reorganization in PSD rats. Furthermore, ta-VNS treatment significantly improved synaptic ultrastructure and upregulated the expression of synapse‐associated proteins. Critically, ALK5 silencing was found to partially attenuate these effects, indicating that the ALK5 signaling pathway underlies the ability of ta-VNS to enhance neuroplasticity.

It has been well established that TGF-β binding to ALK5 induces Smad2/3 phosphorylation. Subsequently, the phosphorylated Smad2/3 forms a heteromeric complex with Smad4, which translocates into the nucleus to orchestrate gene transcription (29, 30, 49, 50). The Gadd45 family comprises three members: Gadd45a, Gadd45b, and Gadd45y. Among these, Gadd45b is uniquely regulated by TGF-β (51, 52). Functioning as a stress-response gene, Gadd45b is activated by various physiological and environmental stressors. It plays important roles in apoptosis, cell-cycle arrest, cell growth, and DNA repair (53). In particular, Gadd45b contributes to BDNF promoter demethylation by enabling the elimination of 5HMC. Neuroplasticity is governed by substances like BDNF, which is absent in patients with depression. Tan et al. identified Gadd45b as a potential therapeutic target to promote adult neurogenesis after cerebral ischemia (54). Recent research has identified Gadd45b as a promising drug candidate for treating PSD (55). Our results demonstrate that the expression changes in the ALK5/Smad2/3 signaling pathway are consistent with those of Gadd45b at all time points. However, when viral intervention was applied to inhibit ALK5 expression, the expression of its downstream signaling molecules and Gadd45b subsequently decreased. Upregulation of the ALK5/Smad2/3/Gadd45β pathway correlated with improvements in neural plasticity and depressive-like symptoms, suggesting that ALK5 functioned upstream of Gadd45b. The Co-IP results indicated an interaction between the two proteins. This confirmed the previously hypothesized link between ALK5/Smad2/3 and Gadd45β.

This study has several limitations. First, the validation of the overexpressed ALK5 signaling pathway and its investigation in established in vitro models remain to be established and constitute key directions for our ongoing research. Second, future research should investigate whether different frequencies and durations of vagus nerve stimulation exert distinct effects on neuroplasticity in the PFC. It is crucial to determine this, as such differences could lead to divergent or even opposing outcomes in the treatment of PSD. Furthermore, as signaling pathways do not act in isolation, our preliminary experiments suggests that vagus nerve stimulation may regulate the ALK5-related signaling pathway by activating the cholinergic anti-inflammatory pathway. This aspect requires further investigation in the future. Finally, future studies in female rats, particularly those mimicking the postmenopausal state (via ovariectomy or aged females), are urgently needed to translate our findings to the clinical setting where PSD is most severe in women. Despite these constraints, this study provides novel insights into the pathological mechanisms of PSD and identifies new therapeutic targets. It also proposes a non-pharmacological ta-VNS approach for clinical management, which avoids the side effects associated with pharmaceuticals and offers a distinct treatment strategy.

## 5 Conclusion

In summary, this study provides compelling evidence that ta-VNS is an effective treatment for PSD. Its efficacy can be attributed to the promotion of neuroplasticity in the PFC, a brain region where impaired plasticity plays a pivotal role in the pathogenesis and progression of PSD. We further demonstrate that ta-VNS alleviates depressive-like behaviors by restoring PFC neuroplasticity, a process likely mediated through the activation of the ALK5/Smad2/3/Gadd45β signaling pathway. These findings hold promise for developing new PSD treatments by revealing novel molecular targets and paving the way for future research.

## Data Availability

The raw data supporting the conclusions of this article will be made available by the authors, without undue reservation.
